# Retinoids enhance NK effector function against HIV-infected CD4 T cells

**DOI:** 10.1128/jvi.01620-25

**Published:** 2026-01-27

**Authors:** Elyse K. McMahon, Jonathan S. Lochner, Rebecca M. Lynch, Alberto Bosque

**Affiliations:** 1 Department of Microbiology, Immunology and Tropical Medicine, George Washington University, Washington, DC, USA; Icahn School of Medicine at Mount Sinai, New York, New York, USA

**Keywords:** NK, natural killer cells, retinoids, HIV, IL-15, HLA-F, KIR3DS1

## Abstract

Novel approaches to sensitize latently infected cells to apoptosis may provide additional methods to eliminate latent reservoirs. Prior research identified several retinoids as potential drugs that increase the sensitivity of HIV-infected cells to cell death. Retinoids are derivatives of vitamin A that target retinoid receptors causing antiproliferative and proapoptotic activity. Several are FDA-approved or in clinical trials. The aim of this study was to evaluate the ability of vitamin A, three of its natural metabolites, and nine synthetic derivatives to sensitize HIV-infected CD4 T cells to NK natural cytotoxicity and antibody-dependent cellular cytotoxicity (ADCC). From the retinoids tested, alitretinoin, tazarotene acid, and AM80 significantly enhanced NK natural cytotoxicity in the presence of IL-15. Mechanistically, these retinoids increased NK degranulation upon target recognition in an HLA-F/KIR3DS1-dependent manner. Furthermore, these retinoids enhanced ADCC by transcriptionally increasing CD16 expression on NK cells. In conclusion, our study has identified at least three retinoids capable of enhancing NK natural cytotoxicity and ADCC against HIV-infected cells. These or other retinoids could be used to reduce HIV persistent reservoirs.

**IMPORTANCE:**

This study highlights how retinoids, compounds derived from vitamin A, can help the immune system target HIV-infected cells more effectively. HIV often hides in immune cells, making it difficult to fully eliminate the virus. We found that certain retinoids, including alitretinoin, tazarotene acid, and AM80, improve the function of natural killer (NK) cells—key immune cells that target infected cells. These retinoids boost NK cell activity by increasing their ability to release toxic molecules that kill infected cells and by enhancing their response to antibodies targeting HIV. This makes the infected cells more vulnerable to being eliminated. Since some of these retinoids are already approved for medical use, they could offer a promising way to reduce persistent HIV reservoirs in the body and improve efforts to cure the infection.

## INTRODUCTION

Human immunodeficiency virus (HIV) causes a global pandemic and requires daily administration of antiretroviral therapy (ART) to suppress virus replication and prevent progression to acquired immunodeficiency syndrome (AIDS). However, interruption of ART results in viral rebound from a subset of cells harboring integrated replication-competent provirus (1–3). While there have been many advances in ART regimens against HIV, a generalized cure has not yet been achieved. Therapeutics have been focused on a variety of methodologies to eradicate the latent reservoir, with many focusing on the shock and kill strategy. With this approach, latently infected cells are reactivated with a latency-reversing agent (LRA) and then killed by either viral cytopathic effects (4–6) or by cellular immune-mediated clearance (7, 8). While several LRAs have been characterized to date, less research has been done in identifying small molecules to enhance immune-mediated strategies to better eradicate latent reservoirs.

Cell-based immune therapies are a growing field within HIV. While many immune therapies focus on improving CD8 T cell responses, there is sufficient evidence that natural killer (NK) cells play an equally important role in immune surveillance and control of HIV infection (9–11). In acute HIV infection, there is a rapid expansion of cytotoxic NK cells (12, 13), but as the infection progresses to the chronic stage, less functional subsets accumulate, leading to deteriorated NK cell function (13–17). Once ART is initiated, NK cell effector function is either restored to normal levels or remains unchanged (10). In general, cytotoxic NK cell functions are suppressed in ART-treated people living with HIV (PWH) when compared to HIV-negative individuals, despite continuous NK cell activation (18, 19). While NK cells are effective at initial infection, they are still limited in their ability to fight off viral infection over a prolonged period of time.

Enhancing NK cell effector functions may provide another avenue to eradicate HIV-infected cells. IL-15 is a gamma-c (γc)-cytokine critical for NK cell development, maturation, survival, proliferation, and cytotoxic function (20–22). IL-15, or its superagonist N-803 (referred to as Anktiva), has been used both *in vitro* as an LRA (8, 23, 24) and in clinical trials (25, 26) to determine its safety in PWH. Previous studies from us and others have shown that IL-15 also enhances NK cell ability to kill HIV-infected CD4 T cells (22, 23, 27, 28). However, the effects of IL-15 in clinical trials have been shown to be modest with a small but significant reduction in the frequency of cells with an inducible HIV provirus (26). Furthermore, the benefits of IL-15 can be impeded by the upregulation of antiapoptotic proteins in infected cells (29, 30). As such, there is an interest in discovering strategies to enhance IL-15-mediated NK killing of HIV-infected cells.

During acute HIV infection, there is an expansion of cytotoxic (CD56^dim^CD16^+^) NK cells, while there is a depletion of cytokine-producing NK cells (CD56^bright^, CD16^−^) (9, 17, 31). Cytotoxic NK cells express the FcγRIIIA (CD16) receptor that binds the Fc domain of IgG antibodies, mediating antibody-dependent cellular cytotoxicity (ADCC) (9). Specifically, the Fc portion of a broadly neutralizing antibody (bNAb) binds to the CD16 receptor on NK cells, and the Fab portion identifies the target cell, which then results in downstream signaling pathways ending with enhanced cytokine release, degranulation, and cytotoxicity (32–34). bNAbs are well studied in the context of HIV (32, 35–38). bNAbs have been shown to exert significant immune pressure on the virus (39, 40) and reduce viral rebound during analytic treatment interruption (ATI) (41, 42). However, similar to IL-15, bNAbs are not sufficient to fully eradicate the reservoir, and further studies are warranted to evaluate strategies to enhance the effector function of bNAbs.

One area that has been expanding is the use of retinoids to promote latency reversal and apoptosis of latently infected cells (43–46). Additionally, retinoids have been used broadly in both cancer treatments (47–49) and skin conditions (50, 51), as they promote apoptosis of cancer cells and sebocytes, respectively. Retinoids are derivatives of vitamin A and can have pleiotropic effects by activating nuclear receptors resulting in proliferation and apoptosis. There are nine nuclear receptors for retinoids: retinoic acid receptor (RAR) α, β, and γ; retinoid X receptor (RXR) α, β, and γ; and RAR-related orphan receptor (ROR) α, β, and γ. We have previously found that isotretinoin, or 13-cis retinoic acid, enhances IL-15-mediated latency reversal and sensitizes these reactivated CD4 T cells to apoptosis (46). Based on this, we hypothesize that retinoids can also enhance NK killing of HIV-infected CD4 T cells. In this work, we evaluated the ability of vitamin A, three of its natural metabolites, and nine synthetic derivatives to enhance NK effector function against HIV-infected CD4 T cells. We showed that retinoids enhance NK natural cytotoxicity and ADCC of HIV-infected CD4 T cells by complementary mechanisms. These results show that retinoids could be used either alone or in combination to target HIV latent reservoirs and add to the arsenal of tools toward finding an HIV cure.

## RESULTS

### Retinoids enhance IL-15-mediated NK killing of infected CD4 T cells

**Fig 1:**
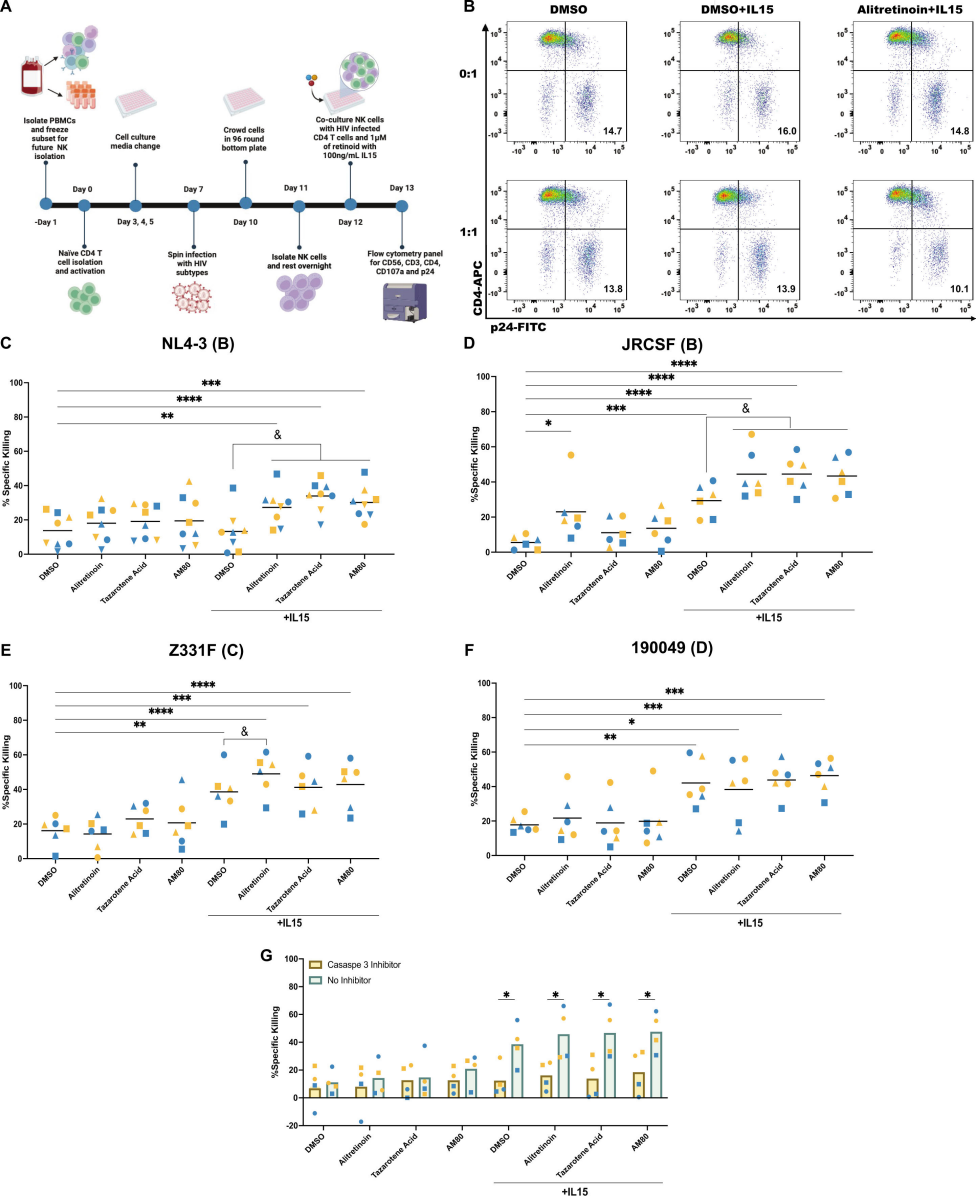
Retinoids enhance IL-15-mediated NK killing of HIV-infected CD4 T cells. (**A**) Timeline of experimental design and representative flow plot (**B**). (**C**) Analysis of percent specific killing of HIV_NL4-3_-infected CD4 T cells in the absence or the presence of IL-15 with the addition of 1 μM of each retinoid. Analysis of percent specific killing for JRCSF (**D**), Z331F (**E**), and 190049 (**F**). Data were normally distributed resulting in parametric analysis. A two-way ANOVA with multiple comparisons was used to determine significance to the DMSO (*P < 0.05, **P < 0.01, ***P < 0.001, ****P < 0.0001) and IL-15 with DMSO (^&^*P* < 0.05, ^&&^*P* < 0.01, ^&&&^*P* < 0.001) (*n* = 8). (**G**) The percent specific killing of HIV_NL4-3_-infected cells in the presence of a caspase-3 inhibitor (*n* = 4). A paired *t*-test was used to determine significance (*P < 0.05). Each participant is designated by their own symbol in each graph. Gold symbols represent male donors, and blue symbols represent female donors.

We have previously shown that the retinoid isotretinoin sensitizes HIV-infected cells to cell death (46). In this study, we wanted to evaluate whether isotretinoin and other retinoid derivatives could also sensitize HIV-infected cells to NK killing. We first evaluated vitamin A, three of its natural metabolites, including isotretinoin, and nine synthetic derivatives for their ability to enhance NK killing of HIV-infected CD4 T cells (Table 1). Briefly, PBMCs were isolated from HIV-negative donors. A fraction of PBMCs was frozen for future NK isolation, and a subset was rested overnight (Fig. 1A). Naïve CD4 T cells were isolated from rested PBMCs and then activated and expanded for 6 days in the presence of IL-2. CD4 T cells were infected via spinoculation at day 7 with the laboratory-adapted strain NL4-3 (subtype B). On day 10, HIV-infected CD4 T cells were crowded to enhance cell-to-cell transmission of the virus. On day 11, NK cells were isolated from autologous frozen PBMCs and rested overnight. The next day, NK cells were co-cultured with autologous HIV-infected CD4 T cells at an effector-to-target (E:T) ratio of 1:1 in the absence or presence of IL-15 (100 ng/mL) and 1 μM of each retinoid for 24 h. NK killing was evaluated by flow cytometry, assessing the reduction in the percentage of productively infected cells (CD4^−^, p24^+^) (Fig. 1B; Fig. S1).

**TABLE 1:** List of retinoids evaluated

Retinoid	Generation	FDA	Type	Targets	References
Vitamin A		Yes	Vitamin	Precursor	(52, 53)
Tretinoin	1st	Yes	Natural metabolite	RARα,β,γ	(54, 55)
Isotretinoin	1st	Yes	Natural metabolite	RARα,β,γ	(56, 57)
Alitretinoin	1st	Yes	Natural metabolite	RAR α,β,γ; RXR α,β,γ	(58, 59)
Acitretin	2nd	Yes	Synthetic	CRABP-I, CRABP-II	(60, 61)
Adapalene	3rd	Yes	Synthetic	RAR α,β,γ	(62, 63)
Bexarotene	3rd	Yes	Synthetic	RXR α,β,γ	(64)
Tazarotene	3rd	Yes	Synthetic	Prodrug	(65, 66)
Tazarotene acid	3rd	Yes	Synthetic	RARβ,γ	(65, 67)
AM80	3rd	No	Synthetic	RARα>RARβ,γ	(68, 69)
Palovarotene	4th	No	Synthetic	RARγ	(70)
Neoruscogenin	4th	No	Synthetic	RORα	(71)
SR0987	4th	No	Synthetic	RORγ	(72)

In the absence of IL-15, none of the retinoids had a significant effect on the ability of NK cells to kill HIV_NL4-3_-infected CD4 T cells over DMSO control (Fig. 1C; Fig. S2A). On the other hand, all retinoids enhanced NK cell-mediated killing in the presence of IL-15, with alitretinoin, tazarotene, its prodrug tazarotene, and AM80 showing the most significant effect (Fig. 1C; Fig. S2B). We then used the Bliss independence model to evaluate the potential synergy of each retinoid with IL-15. This model uses probability to determine if two drugs are acting through independent mechanisms. Values greater than 0 are considered synergistic, values equal to 0 are considered independent, and values below 0 are considered antagonistic (73). Alitretinoin, tazarotene acid and its prodrug tazarotene, and AM80 were all synergistic with IL-15 (Fig. S2C). None of the retinoids presented toxicity at the concentrations tested (Fig. S2D and E). Because of the synergistic effects, we focus the rest of our study on alitretinoin, tazarotene acid, and AM80.

To determine the retinoid’s influence on NK effector function on various HIV subtypes, we evaluated the ability of these three retinoids to enhance killing of HIV-infected cells with different HIV strains including JRCSF (subtype B), Z331F (subtype C), or 190049 (subtype D) (74). Alitretinoin enhances NK killing of HIV_JRCSF_-infected CD4 T cells compared to the DMSO control, and the combination of alitretinoin, tazarotene acid, and AM80 with IL-15 further enhanced NK effector function compared to IL-15 alone (Fig. 1D). Furthermore, IL-15 was able to enhance NK killing of HIV_Z331F_-infected CD4 T cells, and alitretinoin significantly enhanced the ability of IL-15 to promote effector function (Fig. 1E). On the other hand, none of the retinoids, in the absence or presence of IL-15, were able to enhance NK killing of HIV_190049_-infected CD4 T cells over IL-15 alone (Fig. 1F). We have previously shown that the retinoid isotretinoin sensitized HIV-infected cells to cell death via enhanced caspase-3 activation (46). As such, we assessed the contribution of this pathway by measuring specific killing of HIV-infected cells in the presence of the caspase-3/7 inhibitor TF3-DEVD-FMK using our established co-culture model. Inhibition of caspase-3 significantly reduced NK cell-mediated killing of HIV_NL4-3_-infected CD4 T cells in the presence of retinoids and IL-15 (Fig. 1G).

**Fig 2:**
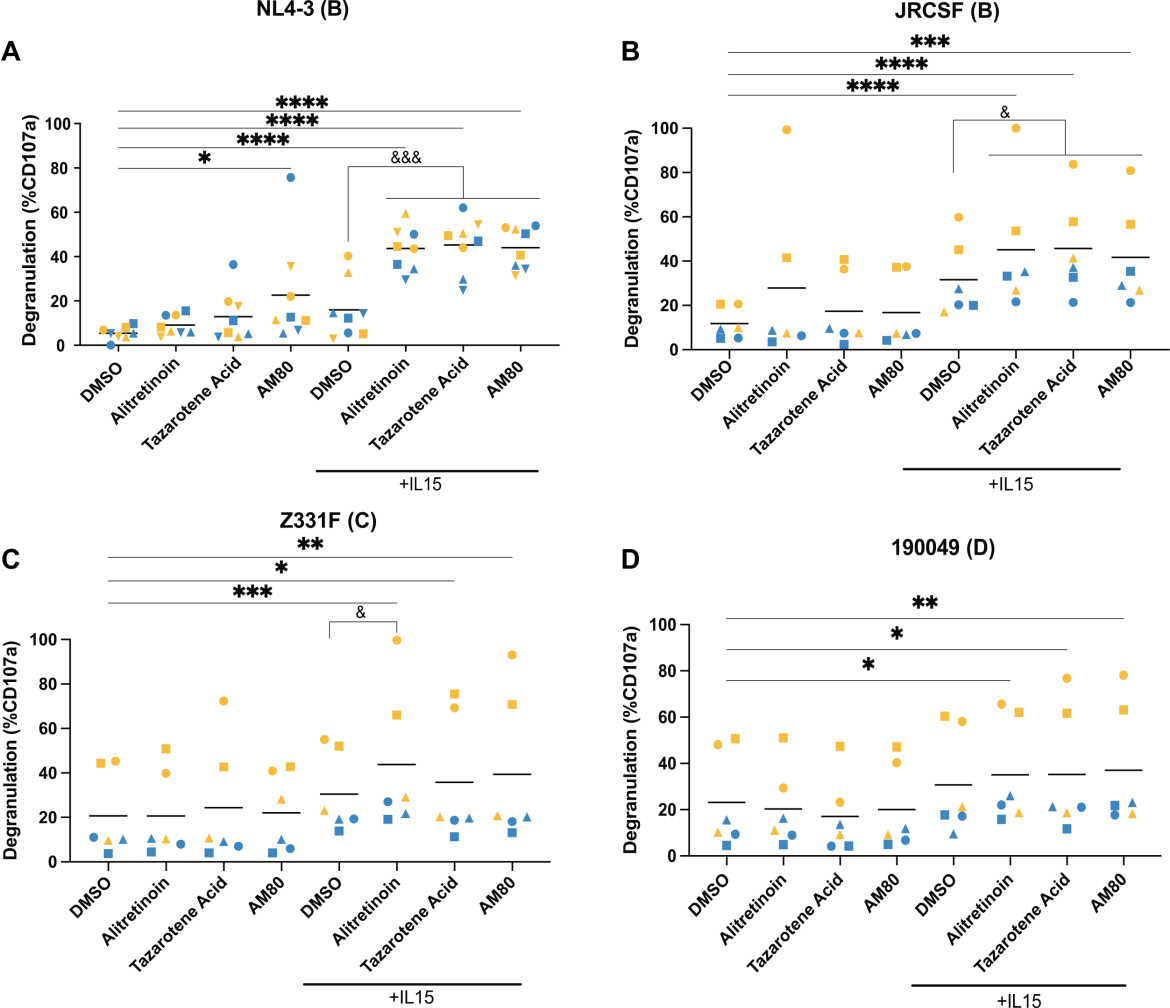
Retinoids enhance IL-15-mediated NK degranulation. Analysis of NK degranulation when NK cells were cultured with or without IL-15 and 1 μM of each retinoid exposed to CD4 T cells infected with (**A**) HIV_NL4-3_, (**B**) HIV_JRCSF_, (**C**) HIV_Z331F_, and (**D**) HIV_190049_ (*n* = 6). A two-way ANOVA with multiple comparisons was used to determine significance relative to the DMSO (*P < 0.05, **P < 0.01, ***P < 0.001, ****P < 0.0001) and IL-15 with DMSO (^&^*P* < 0.05, ^&&&^*P* < 0.001). Each participant is designated by their own symbol in each graph. Gold symbols represent male donors, and blue symbols represent female donors.

To identify potential mechanisms driving the enhanced killing of HIV-infected CD4 T cells by retinoids, we first measured expression of the cytotoxic proteins granzyme A, granzyme B, and perforin, which are associated with NK cytotoxicity. NK cells were cultured in the absence or presence of IL-15 (100 ng/mL) and 1 μM of each retinoid for 24 h (75). Retinoids alone did not enhance the expression of these molecules when compared to DMSO; however, as previously shown, the addition of IL-15 significantly enhanced the expression of all three molecules (Fig. S3) (23, 27, 76, 77). We then measured NK degranulation by assessing surface expression of CD107a on NK cells (Fig. S4A). In agreement with the increase in natural cytotoxicity of HIV_NL4-3_-infected cells, we found that several retinoids enhanced NK degranulation in the presence of IL-15, but not in the absence of IL-15, in a synergistic manner (Fig. 2A; Fig. S4B through D). To confirm that degranulation was associated with target recognition and not due to non-specific activity of the retinoids, we cultured NK cells alone, with uninfected CD4 T cells, or with HIV_NL4-3_-infected CD4 T cells in the presence of the retinoids, either alone or with IL-15. The increase in NK degranulation was only observed in the presence of HIV_NL4-3_-infected CD4 T cells, demonstrating the need for target recognition (Fig. S4E). NK peak degranulation occurs within 4 to 6 h after target recognition (78, 79). We initially measured degranulation at the end of the coculture. We then measured and compared CD107a after 6 or 24 h of co-culture. We found that retinoids, both in the absence and presence of IL-15, enhanced degranulation at 6 h when compared to the DMSO control (Fig. S4F). Interestingly, the combination of retinoids and IL-15 led to sustained degranulation throughout the 24-hour co-culture. Similar degranulation results were observed with the other viral strains (Fig. 2B through D). Next, we evaluated whether there was a correlation between degranulation and cytotoxicity. We observed a significant positive correlation between the percent specific killing of HIV_NL4-3_-infected CD4 T cells and NK degranulation for the three retinoids tested in the presence of IL-15 in both male and female donors (*P* = 0.021 and *P* = 0.015, respectively) (Fig. S5).

Altogether, our results demonstrate that the combination of IL-15 with retinoids may be increasing recognition of HIV-infected CD4 T cells, leading to enhanced NK degranulation and enhanced killing in a caspase-3-dependent manner. This mechanism is conserved among subtypes B and C, but may not be as important for subtype D, for which IL-15 is sufficient to enhance killing.

### IL-15 enhances HLA-F expression in HIV-infected CD4 T cells

**Fig 3:**
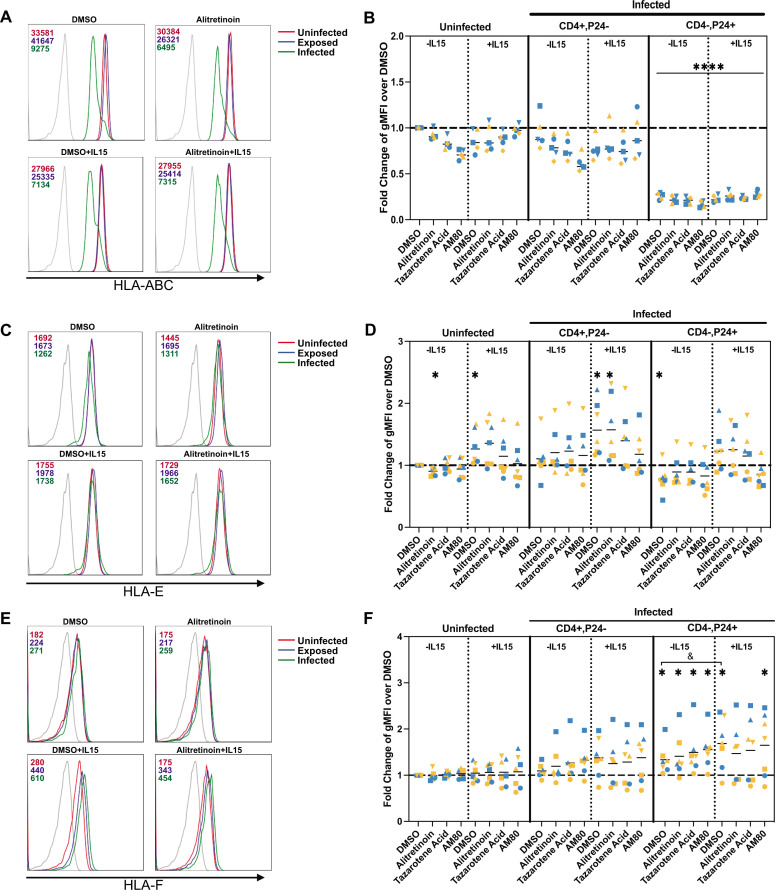
Retinoids do not influence HLA expression in uninfected or HIV-infected CD4 T cells. Fold change expression of geometric mean intensity fluorescence (gMFI) over DMSO of uninfected and infected cells for (**A, B**) HLA-ABC (*n* = 5), (**C, D**) HLA-E (*n* = 7), and (**E, F**) HLA-F (*n* = 7). Data were normally distributed, resulting in parametric analysis. A one-sample *t-*test was used to compare the fold change to the DMSO of uninfected cells (*P < 0.05, ****P < 0.0001), and a paired *t*-test was used to compare the addition of IL-15 to the absence of IL-15 in HIV-infected cells (^&^*P* < 0.05). Each participant is designated by their own symbol in each graph. Gold symbols represent male donors, and blue symbols represent female donors.

As our results suggest that retinoids may enhance the recognition of HIV-infected CD4 T cells, we evaluated different self/non-self signals derived from the interactions between receptors on NK cells and their associated ligands on target cells. We first analyzed the expression of several MHC class I molecules that are influenced by HIV infection, including HLA-A, -B, -C, -E, and -F, all of which have been shown to play a role in NK recognition of HIV-infected cells (80–83). HIV_NL4-3_-infected CD4 T cells were incubated with 1 μM of each retinoid in the absence or presence of IL-15 (100 ng/mL) for 24 h, and then stained for CD4, p24, and either pan-HLA-ABC, HLA-E, or HLA-F antibodies (Fig. S6). We measured MHC-I expression in uninfected cultures and in infected cultures, including both uninfected but exposed (CD4^+^, p24^−^) and productively infected populations (CD4^−^, p24^+^), within the same culture. As expected, HLA-ABC was significantly downregulated in the infected population with respect to both the uninfected population and the exposed, but the presence of retinoids with or without IL-15 did not influence this downregulation (Fig. 3A and B). HLA-E expression was highly variable among donors, with significant upregulation in uninfected cells in the presence of IL-15 and significant downregulation in infected cells without the addition of retinoids, compared to the uninfected control (Fig. 3C and D). Finally, we measured HLA-F expression and found a significant upregulation in infected cells when compared to the uninfected control DMSO (Fig. 3E and F). The addition of IL-15 in infected cells further upregulated the expression of HLA-F compared to the uninfected DMSO control, but retinoids did not contribute to this upregulation. Similar patterns were found for JRCSF (subtype B), Z331F (subtype C), and 190049 (subtype D) (Fig. S7). In conclusion, retinoids did not influence the expression of HLA molecules on the surface of HIV-infected CD4 T cells.

### Retinoids enhance the interaction between NK cells and HIV-infected cells through HLA-F and KIR3DS1

**Fig 4:**
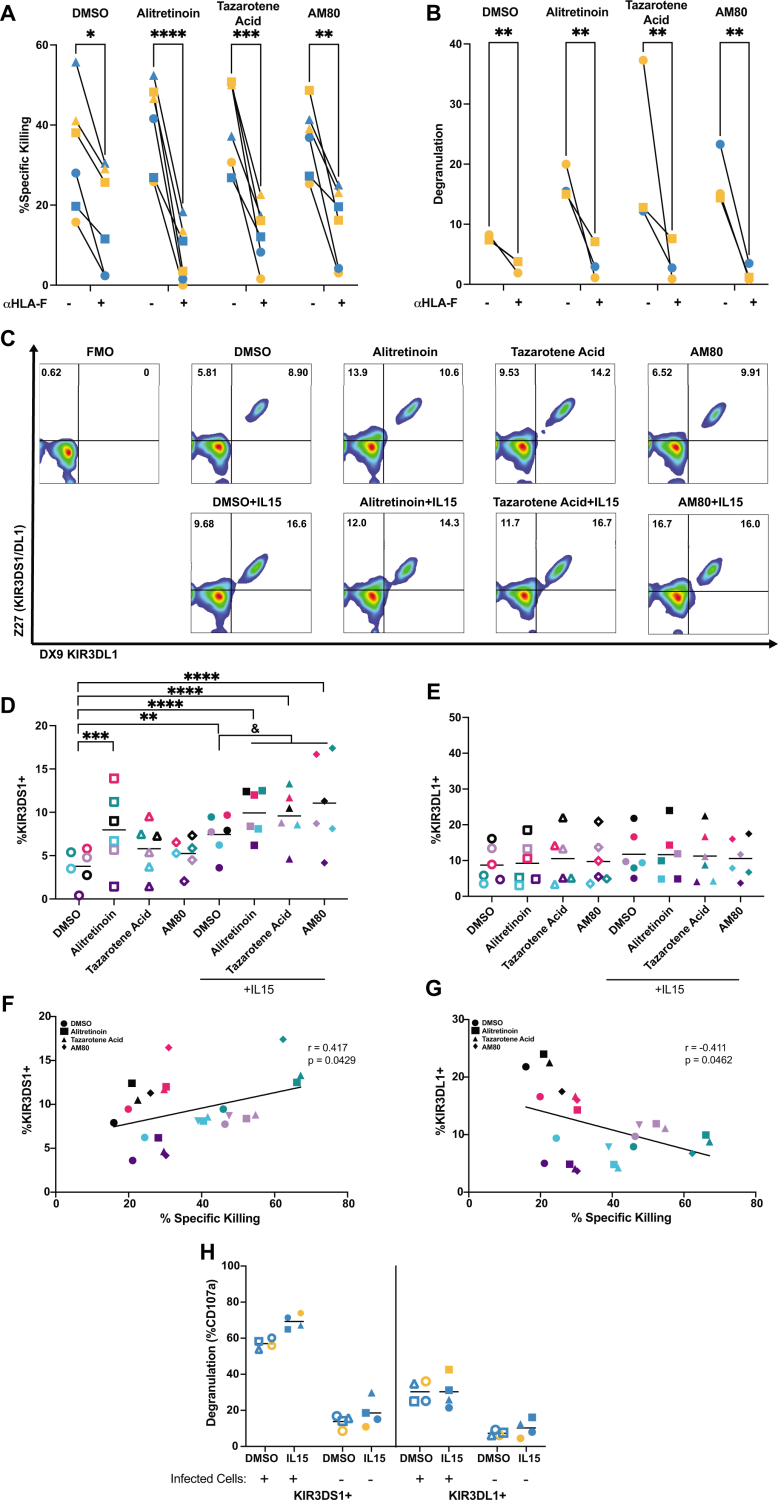
Retinoids enhance HIV killing through increasing KIR3DS1 and HLA-F interaction. (**A**) Analysis of percent specific killing (*n* = 5) and (**B**) NK degranulation (*n*=3) of HIV_NL4-3_-infected CD4 T cells in the absence and presence of the HLA-F blocking antibody. A two-way ANOVA was used to compare with and without the blocking antibody for each condition. NK cells cultured with retinoids in the absence and presence of IL-15 for 24 h to measure (**D**) KIR3DS1^+^ (*n* = 6) and (**E**) KIR3DL1^+^ (*n*=6). Data were normally distributed, resulting in parametric analysis. A one-sample *t*-test was used to compare to the DMSO control, and a RM one-way ANOVA was used to compare the combination of retinoids and IL-15 to IL-15 alone (^&^*P* < 0.05). Analysis of the association between percent specific killing in (**F**) KIR3DS1^+^ (*n* = 6) and (**G**) KIR3DL1^+^ NK cells (*n* = 6). We used a Pearson correlation analysis to determine significance. (*P < 0.05, **P < 0.01, ***P < 0.001, ****P < 0.0001). (**H**) Analysis of NK degranulation in KIR3DS1^+^ and KIR3DL1^+^ NK cells (*n* = 4). Each participant is designated by their own symbol or color in each graph. Gold symbols represent male donors, and blue symbols represent female donors in panels A, B, H, and I. In panels D to G, each color represents an individual participant with open symbols representing the absence of IL-15 and filled-in symbols representing the presence of IL-15.

Based on the observation that IL-15 upregulated HLA-F in productively infected cells, we tested if HLA-F influences NK cell target recognition. Briefly, infected CD4 T cells were treated with 25 μg/mL of purified anti-human HLA-F blocking antibody (clone 3D11) for 50 min at 37°C (83). After this, autologous NK cells were cultured with treated infected cells, and 1 µM of each retinoid with 100 ng/mL of IL-15 was added to the culture for 24 h. Following this, both the percent specific killing and NK degranulation were measured. We observed that cells treated with 3D11 reduced retinoid-mediated NK killing of HIV-infected cells and degranulation (Fig. 4A and B; Fig. S8A). To confirm the specificity of 3D11, HIV-infected CD4 T cells were treated with the 3D11 blocking antibody, an isotype control, and a no antibody control. We found that the 3D11 blocking antibody did reduce the percent specific killing, while the isotype control was similar to the condition with no antibody (Fig. S8B).

Based on this result, we then measured the activating and inhibitory receptors on NK cells that recognize HLA-F: killer cell immunoglobulin-like receptor, three Ig domains and short cytoplasmic tail 1 (KIR3DS1), and long cytoplasmic tail 1 (KIR3DL1), respectively (82, 84). We cultured NK cells with 1 μM of each retinoid in the presence or absence of 100 ng/mL of IL-15 for 24 h either alone or in the presence of target cells. To differentiate KIR3DS1 and KIR3DL1 expression, cells were stained with two antibodies for flow cytometry analysis. The Z27 clone antibody binds to both KIR3DS1 and KIR3DL1, while the DX9 clone antibody specifically binds to KIR3DL1 (Fig. 4C) (85, 86). This dual staining has been shown to distinguish cells expressing KIR3DL1 to those cells expressing only the activating receptor KIR3DS1 (85, 86). As previously reported, we observed differences between donors showing high or low KIR3DL1 expression along with differing KIR3DS1 expression, which varies by the frequency of allelic expression (87–90) (Fig. S9A). We observed that IL-15 significantly enhanced the proportion of KIR3DS1^+^ NK cells, and the addition of retinoids further enhanced this expression (Fig. 4D), while we did not observe any changes in the proportion of KIR3DL1^+^ NK cells (Fig. 4E). Importantly, the increase in the proportion of KIR3DS1^+^ NK cells was only observed in the presence of target cells, suggesting that target recognition is required for KIR3DS1 upregulation (Fig. S9B and C). This agrees with previous studies indicating that NK cells need to be in the presence of target cells to induce expression of activating receptors (82, 91). Interestingly, in the presence of IL-15, we observed a significant positive correlation between the population of KIR3DS1^+^ NK cells and the percent specific killing of HIV-infected cells (Fig. 4F) (*r* = 0.417, *P* = 0.0429), and a concomitant significant negative correlation between the population of KIR3DL1^+^ NK cells and the percent specific killing of HIV-infected cells (Fig. 4G) (*r* = −0.411, *P* = 0.0462). This correlation was not observed in the absence of IL-15 (Fig. S9D and E). Furthermore, NK degranulation upon target recognition was more prominent in KIR3DS1^+^ NK cells than KIR3DL1^+^ NK cells, which aligns with previous studies demonstrating similar results (Fig. 4H) (12, 83).

Finally, we tested other activating and inhibiting ligands on NK cells. We first measured changes in the expression of natural killer group 2 members (NKG2), including NKG2A, NKG2C, and NKG2D, upon culturing NK cells with the retinoids, in the absence or presence of IL-15, in both CD56^bright^CD16^−^ and CD16^+^ subpopulations. While IL-15 alone modestly increased the expression of certain receptors, retinoids did not modify their expression (Fig. S10). Together, these results demonstrate that retinoids exert their activity by increasing the interaction between HLA-F and KIR3DS1, leading to enhanced degranulation and killing of HIV-infected cells.

### Retinoids enhance ADCC by promoting CD16 expression

**Fig 5:**
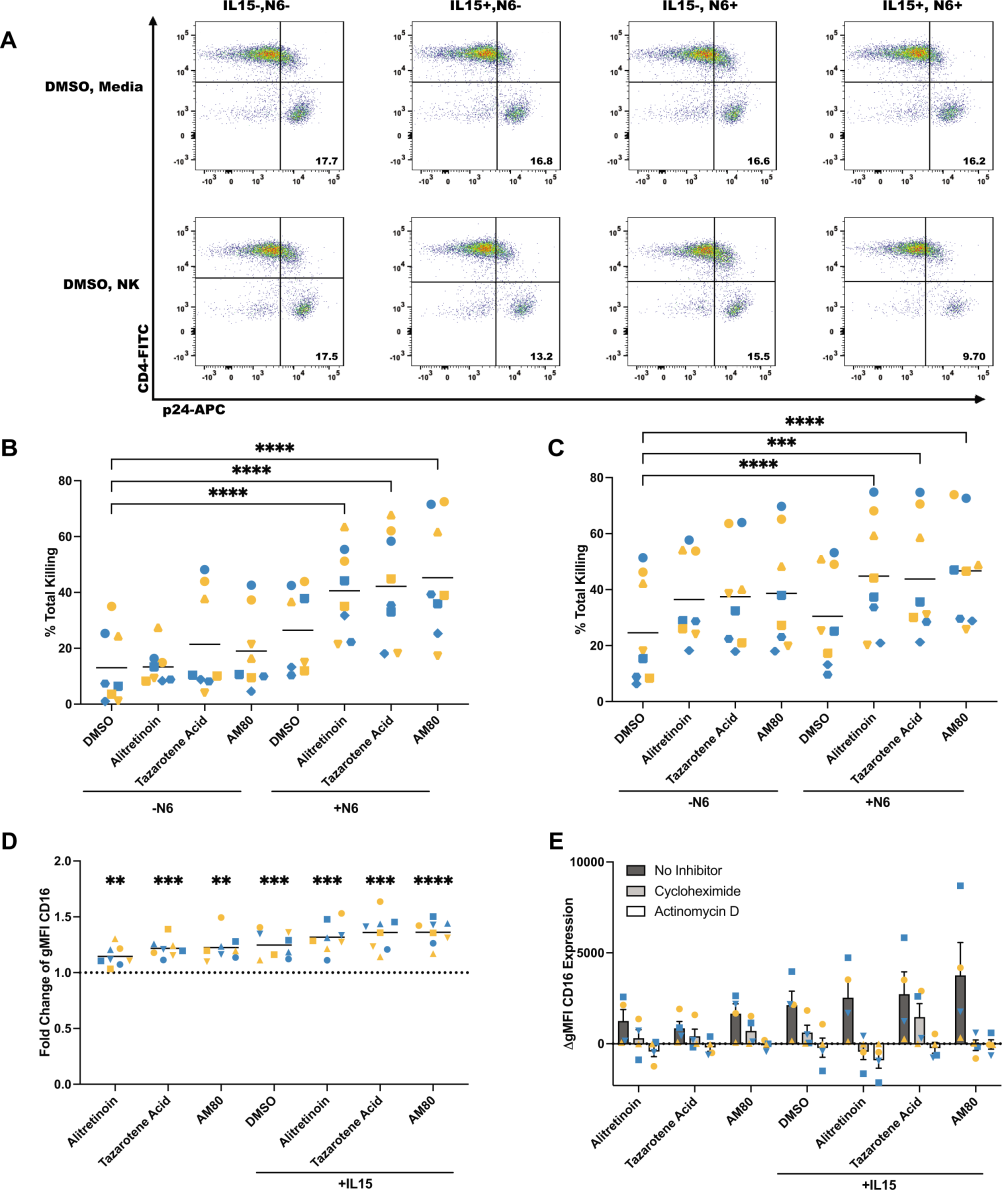
Retinoids enhance antibody-dependent cellular cytotoxicity via transcriptional enhancement of CD16 expression. (**A**) Representative flow plot. Analysis of percent specific killing of HIV_NL4-3_-infected CD4 T cells with the addition of the N6 antibody and retinoids in the absence (**B**) or presence (**C**) of IL-15 (*n* = 8). Data were not normally distributed, resulting in non-parametric analysis. The Friedman test was used to compare the percent total killing in the presence of the N6 antibody to the DMSO control in the absence of the N6 antibody. (**D**) Fold change in CD16 expression on NK cells over the DMSO control with the addition of retinoids with or without IL-15 (*n* = 8). A one-sample test was used to compare the fold change values to the DMSO control. (*P < 0.05, **P < 0.01, ***P < 0.001, ****P < 0.0001). (**E**) Change in CD16 expression on NK cells cultured with the indicated retinoids in the absence or presence of IL-15 and the transcriptional inhibitor actinomycin D or the translational inhibitor cycloheximide (*n* = 4). Each participant is designated by their own shape in each graph. Gold symbols represent male donors, and blue symbols represent female donors.

Based on the results demonstrating that retinoids enhance natural cytotoxicity, we next wanted to evaluate whether retinoids would influence ADCC. To measure ADCC, we conducted similar experiments using HIV_NL4-3_-infected primary CD4 T cells co-cultured with autologous NK cells at an E:T ratio of 1:1, in the presence of the bNAb N6 (Fig. 5A). The combination of retinoids with the N6 antibody significantly enhanced the amount of NK killing of infected CD4 T cells compared to DMSO control both in the absence (Fig. 5B) and in the presence of IL-15 (Fig. 5C). Similar results were obtained at an E:T ratio of 2:1 (Fig. S11A and B). To determine if HLA-F is involved in ADCC as it is for natural cytotoxicity, we performed additional HLA-F blocking experiments as described above. As expected, we found that blocking HLA-F does not influence ADCC (Fig. S11C). To further understand the potential mechanisms associated with enhanced ADCC mediated by retinoids, we focused on the Fc receptor CD16 on NK cells, as it is required for ADCC (31). Interestingly, CD16 expression is predominantly in KIR3DL1^+^ NK cells, but retinoids and IL-15 increase CD16 expression in both populations (Fig. S11D). These results suggest that KIR3DS1^+^ NK cells may play a more important role in natural cytotoxicity, while KIR3DL1^+^ NK cells may play a role in ADCC. We observed that retinoids are sufficient to enhance CD16, and this increase is enhanced in the presence of IL-15 (Fig. 5D). To elucidate the mechanisms by which retinoids induce CD16 expression, we used either the transcriptional inhibitor actinomycin D (ActD) or the translational inhibitor cycloheximide (CHX). Both inhibitors blocked retinoid-mediated CD16 induction in primary NK cells (Fig. 5E; Fig. S12). These results suggest that retinoids are regulating CD16 expression in NK cells at the transcriptional level, leading to enhanced ADCC.

## DISCUSSION

In this study, we examined the potential role of retinoids in enhancing NK effector function against HIV-infected CD4 T cells. We and others have previously identified different retinoids that sensitize HIV-infected CD4 T cells to cell death (43, 46). Since several retinoids are FDA-approved or in clinical trials, we evaluated their ability to enhance NK effector functions against HIV, particularly natural cytotoxicity and ADCC. Our results demonstrate that several retinoids are sufficient to synergize with IL-15 to promote natural cytotoxicity and enhance ADCC.

Of the 13 retinoid derivatives tested, we found that alitretinoin, tazarotene acid, and AM80 consistently enhance NK function. Mechanistically, our data demonstrate that inhibition of caspase-3 significantly attenuated NK-mediated killing, supporting a model in which retinoids sensitize HIV-infected targets to apoptosis through a caspase-dependent pathway (46, 92). Alitretinoin is a first-generation, FDA-approved natural derivative of vitamin A and has been used in treatments of T cell lymphoma (93, 94), as well as skin conditions, such as dermatitis and eczema (95). This retinoid primarily binds to six of the retinoid receptors, including RAR α, β, and γ, along with RXR α, β, and γ (58, 59). Tazarotene acid is a third-generation FDA-approved synthetic retinoid used in topical treatments (96), along with early skin cancer clinical trials (97, 98). It is the active metabolite of tazarotene, which has been used to treat a variety of skin conditions (99). It primarily targets RAR β and γ (65, 67). Finally, AM80, also known as tamibarotene, is not yet FDA-approved in the United States but has entered clinical trials for treating pancreatic cancer (NCT05064618). It is currently approved in Japan for acute promyelocytic leukemia (100, 101). It preferentially targets RAR α and has a lower affinity for β and γ (68, 69). Our results suggest that retinoids may exert their function through RAR, but further studies will be warranted to specifically determine which receptor(s) are involved in enhancing NK effector function.

Notable subtype-specific differences were observed regarding the effects of retinoids, underscoring the heterogeneity in susceptibility of HIV strains to NK cell responses. Subtype B strains (NL4-3 and JRCSF) demonstrated pronounced sensitivity to the combination of retinoids and IL-15. These findings align with prior reports indicating that subtype B is more sensitive to immune pressure and NK recognition (102). In contrast, subtype C (Z331F) exhibited a more modest response, with significant enhancement observed only upon the combination of IL-15 and alitretinoin, consistent with previous literature reporting variable immune evasion mechanisms in subtype C viruses (103, 104). Subtype D (190049) remained largely resistant to retinoid-induced enhancement, although IL-15 alone was sufficient to enhance cytotoxic effects, reflecting the inherent differences in immune recognition among HIV clades (105).

**Fig 6:**
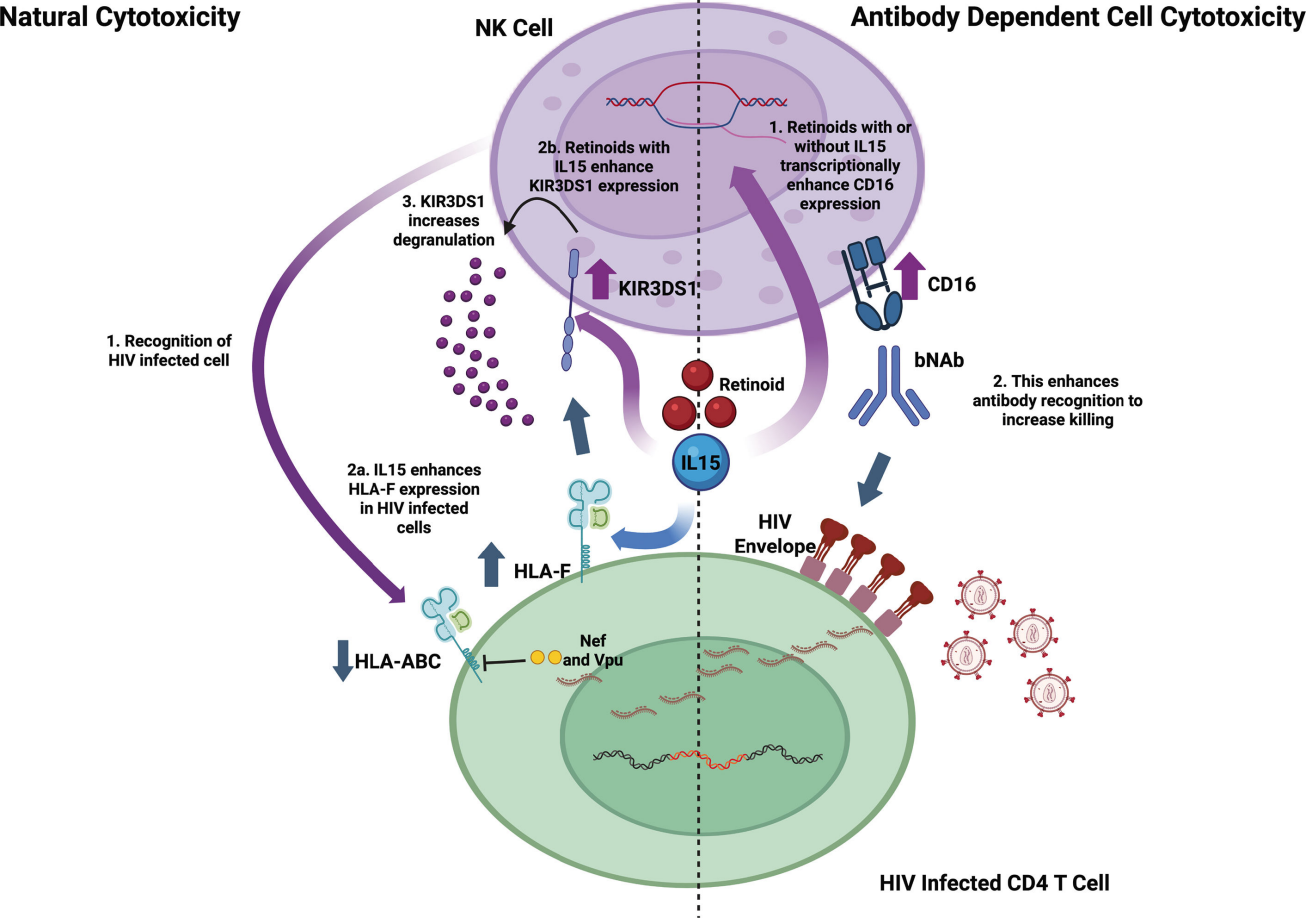
Proposed mechanism of action of retinoids. Natural cytotoxicity: within a co-culture. (1) NK cells recognize infected cells by the downregulation of HLA-ABC. (2a) The addition of IL-15 enhances HLA-F expression which also signals to the ligand KIR3DS1. Simultaneously, (2b) the combination of the retinoids with IL-15 enhances the proportion of KIR3DS1 expression resulting in (3) increased degranulation of NK cells and enhanced killing of HIV-infected cells. Antibody-dependent cell cytotoxicity: (1) retinoids with or without IL-15 enhance CD16 expression at the transcriptional level. (2) The enhanced CD16 expression results in enhanced antibody recognition and increased killing of HIV-infected CD4 T cells by ADCC.

While retinoids did not alter MHC-I expression, we did observe that IL-15 influences some of these molecules. IL-15 promotes CD4 T cell activation, proliferation, survival, and effector function, which are often associated with MHC class I expression (106, 107). However, direct evidence for IL-15-mediated regulation of HLA-ABC on CD4 T cells remains limited. In our study, short-term treatment with IL-15 did not significantly influence HLA-ABC on either infected or uninfected CD4 T cells. In contrast, IL-15 dynamically regulated non-classical MHC class I molecules, HLA-E and HLA-F. IL-15 increased HLA-E expression on uninfected CD4 T cells, whereas infected cells did not exhibit this induction. Notably, untreated infected cells showed a reduction in HLA-E expression, suggesting infection-associated downregulation. Literature remains divided on whether HIV infection upregulates (108, 109) or downregulates (110, 111) HLA-E potentially due to the instability of the molecule on HIV-infected cells (112). On the other hand, IL-15 upregulated HLA-F on HIV-infected cells. HLA-F is less well characterized, but is known to function as an intracellular chaperone and regulates immune function by interacting with activating and inhibitory receptors on effector cells (113, 114). The surface expression is induced under inflammatory conditions, indicating a role in modulating immune responses during viral infections, including HIV (115). With IL-15 further upregulating HLA-F in infected cells, this could represent a novel pathway to enhance NK killing by triggering activation ligands. Notably, blockade of HLA-F significantly reduced NK cell-mediated killing and degranulation in response to HIV-infected cells, suggesting that HLA-F contributes to effective NK cell recognition and activation. These findings align with prior studies showing that HLA-F, especially in its open conformer, serves as a ligand for NK cell receptors such as the activating receptor, KIR3DS1, and the inhibitory receptor, KIR3DL1 (82, 84). KIR3DS1 promotes NK cell activation, leading to IFN-γ production, degranulation, and release of granzymes and perforin (13, 85, 116, 117). Both KIR3DS1 and KIR3DL1 alleles are encoded on the KIR3DL1 locus and can be subdivided based on the extent of their expression on the cell surface (88, 90, 118). To better differentiate these two receptors, previous studies use the combination of the monoclonal antibodies DX9, which only binds to KIR3DL1, and Z27, which binds to both KIRs (85, 86). We observed that the combination of IL-15 and retinoids enhanced KIR3DS1 expression, while not influencing KIR3DL1 expression, but this increase was only observed in the presence of HIV-infected cells. Although there were differences in the expression of KIR3DS1 and KIR3DL1 among the donors tested, we did observe that killing of HIV-infected CD4 T cells was positively correlated with the proportion of KIR3DS1^+^ NK cells and, conversely, negatively correlated with the proportion of KIR3DL1^+^ NK cells. Based on these results, we propose the following mechanism: (1) HLA-ABC, a classical HLA-I molecule, is downregulated in HIV-infected CD4 T cells through Nef and Vpu (80, 81). This is recognized by NK cells. (2a) HLA-F expression is enhanced by the addition of IL-15, which is recognized by NK cells through the KIR3DS1 activating receptor. (2b) KIR3DS1 expression is further enhanced by the combination of IL-15 with the retinoids. (3) This results in enhanced degranulation from KIR3DS1^+^ NK cells and enhanced killing of HIV-infected CD4 T cells in a caspase-3-dependent manner (Fig. 6, left panel).

Besides natural cytotoxicity, NK cells can exert ADCC through the interaction of their FcRγIII (CD16) with the Fc portion of antibodies. Several bNAbs are currently under clinical investigation for their potential to promote clearance of the latent reservoirs, but their effect to date has been minimal (41, 42). As such, identifying strategies that also enhance ADCC by manipulating NK cells could lead to better outcomes. Several bNAbs have been studied in combination (34, 119–121), and a recent clinical trial is evaluating the combination of bNAbs with IL-15 (NCT04340596). To our knowledge, there are no studies that have analyzed the combination effects of retinoids with bNAbs. In this study, we demonstrate that retinoids can enhance ADCC by upregulating CD16 at the transcriptional level (Fig.6, right panel) independently of HLA-F. However, further experiments need to be conducted to fully elucidate the mechanisms associated with increased transcription of CD16.

Our study has several caveats. First, while Kiani and colleagues were able to use both an HLA-F blocking antibody along with a KIR3DS1 blocking antibody (83), we could not block KIR3DS1 ligand due to product discontinuation and lack of availability of alternative antibodies. However, our results demonstrate that blocking HLA-F reverses the increased cytotoxicity observed with the combination of IL-15 and retinoids. Further studies will be required to evaluate the role of KIR3DS1 in this process. Second, our ADCC studies only include the bNAb N6. N6 is one of the most clinically advanced bNAbs that targets the highly conserved CD4 binding site (122). Unlike other previously identified CD4 binding site bNAbs, N6 has a potential neutralization capacity of up to 98% across a large panel of different HIV subtypes, with a low dose requirement and minimal self-epitope binding (122). Third, throughout all co-culture experiments, we isolated NK cells from frozen PBMCs. While this is not ideal to measure both natural and antibody-mediated cytotoxicity due to CD16 being sensitive to cleavage from thawing (123), functional assays using these cryopreserved cells remain valid and reproducible (124). Fourth, throughout the study, all donors were heterozygous for KIR3DS1 and KIR3DL1. While using homozygous KIR3DS1 would have further clarified the interaction between KIR3DS1 and HLA-F in these experiments, the proportion of people that are homozygous for KIR3DS1 in the western hemisphere is small (125–128). Despite these findings, this is the first study, to our knowledge, that extensively evaluates the combination of retinoids and IL-15 to manipulate both natural cytotoxicity and ADCC in NK cells against HIV-infected CD4 T cells. Additionally, it further evaluates the role of HLA-F and KIR3DS1 in NK cell biology and HIV infection. The use of retinoids has been prevalent in many different immune therapy fields, especially within cancer treatments. Our results further support the use of retinoids to be considered within the context of HIV cure approaches, either alone or in combination with other strategies to eliminate latent reservoirs.

## MATERIALS AND METHODS

### Reagents

IL-2 and IL-15 were provided by the BRB/NCI Preclinical Repository. We obtained the following reagents through the NIH HIV Reagent Program, Division of AIDS, NIAID, NIH: nelfinavir, raltegravir, and human immunodeficiency virus 1 (HIV-1), strain JR-CSF infectious molecular clone (pYK-JRCSF), ARP-2708, contributed by Dr. Irvin S. Y. Chen and Dr. Yoshio Koyanagi; human immunodeficiency virus type 1 Z331F infectious molecular clone (SGA 11), ARP-13249, contributed by Dr. Eric Hunter; and human immunodeficiency virus 1 (HIV-1), strain NL4-3 infectious molecular clone (pNL4-3), ARP-114, contributed by Dr. M. Martin. The HIV-1 primary isolate p190049 was a gift from Beatrice H. Hahn. Retinoids were used at a concentration of 1 μM. Isotretinoin was purchased from Selleck Chemicals (cat# S1379). All other retinoids were purchased from Cayman Chemical. This included vitamin A (cat# 202410), tretinoin (cat# 11017), alitretinoin (cat# 14587), acitretin (cat# 20853), adapalene (cat# 13655), bexarotene (cat# 11571), tazarotene (cat# 23559), tazarotene acid (cat# 21367), AM80 (cat# 71770), palovarotene (cat# 28460), neoruscogenin (cat# 15567), and SR0987 (cat# 19503). Dynabeads Human T-Activator CD3/CD28 (cat#11131D) were purchased from ThermoFisher.

### Sex as a biological variable

Throughout this study, we recognized that sex may influence the results. To ensure equal representation, each experiment had an equal number of male and female donors. We conducted further analyses to determine if sex has influenced any of the results we observed.

### PBMCs

Buffy coats were obtained from HIV-negative donors from the Gulf Coast Regional Blood Center. PBMCs were isolated from buffy coats by Lymphoprep cell gradient centrifugation (STEMCELL Technologies, cat#07851). After washing three times in PBS + EDTA (2 mM), the PBMCs were resuspended in RPMI 1640 medium with 10% FBS (Gibco), 1% L-glutamine, and 1% Penicillin/Streptomycin (Gibco). This is referred to as complete media.

### Co-culture experiments

From isolated PBMCs, a subset was rested overnight, while the rest were viably frozen at −80C and then stored in liquid nitrogen for future NK isolation. Naïve CD4 T cells were isolated using the EasySep Human Naïve CD4+ T Cell Enrichment Kit (Stem Cell, cat# 19555) from the rested PBMCs. Cells were activated at 0.5 × 10^6^ cells/mL with αCD3/CD28 Dynabeads in the presence of αIL-4, αIL-12, and TGF-β in complete media for 3 days. On day 3, Dynabeads were removed, and 1 × 10^6^ cells/mL were cultured in complete RPMI with IL-2, replacing the media and IL-2 at day 4 and day 5. On day 7, the cells were infected by spinoculation with either the laboratory-adapted strain NL4-3 or with different HIV strains, including JRCSF, Z331F, or 190049. On day 10, the cells were crowded in 96-well round-bottom plates to enhance transmission of the virus and increase infection rate. On day 11, NK cells were isolated from the frozen PBMCs using the EasySep Human NK Cell Isolation Kit (Stem Cell, cat# 17955) and rested overnight. On day 12, CD4 T cells were uncrowded and then co-cultured with NK cells at a 0:1 or 1:1 E:T ratio in the presence of ART (raltegravir 1 μM and nelfinavir 0.5 μM). The cells were treated based on the experiment being conducted. For co-cultures measuring natural cytotoxicity, the cells were cultured with 1 μM of each retinoid in the absence or presence of 100 ng/mL of IL-15. For co-cultures measuring the influence of caspase-3, TF3-DEVD-FMK caspase-3/7 reagent (AAT Bioquest, cat# 20101) was then added to all cells (1:150). For co-cultures measuring infection rates when HLA-F is blocked, the infected CD4 T cells were treated with 25 μg/mL of purified anti-human HLA-F blocking antibody (clone 3D11, BioLegend, cat# 373202) or the purified mouse IgG1 isotype control antibody (clone MOPC-21, BioLegend, cat# 400101) for 50 min at 37°C. The cells were then co-cultured with NK cells and treated, as previously described. For co-cultures to measure ADCC, the cells were cultured with the same concentration of retinoids and IL-15, along with 1 μg/mL of N6 at a 1:1 or 2:1 E:T ratio.

After the cells were incubated at 37°C for 24 h, the cells were stained to measure HIV infection via flow cytometry. A minimum of 2 × 10^5^ cells was used in staining procedures. The cells were washed with FACS buffer (PBS with 2% FBS and 2 mM EDTA). We stained the cells with 1:100 dilutions of eBioscience Fixable Viability Dye eFluor 450 (Thermo Fisher Scientific, cat# 65-0863-18) with 100 μL of FACS and then incubated at 4°C for 10 min After this, the cells were washed with FACS buffer. Human BD Fc Block (BD BioSciences, cat# 564220) was added, and the cells were incubated at room temperature for 10 min. Following this, the cells were stained with 1:100 dilution of the antibodies in FACS buffer, including anti-human CD4 APC (clone S3.5, ThermoFisher, cat# 17-0048-42), anti-human CD3 BV786 (clone SP34-2, BD BioSciences, cat# 563918), anti-human CD56 PercP-Cy5.5 (clone 5.1H11, BioLegend, cat# 362506), anti-human CD107a PE (clone H4A3, Southern Biotech, cat# 9835-09), anti-human KIR3DS1/L1 PE (clone Z27.3.7, Beckman Coulter, cat# IM3292), and/or anti-human KIR3DL1 BV421 (clone DX9, BioLegend, cat# 312714). After a 30-minute incubation at 4°C, the cells were washed and then fixed and permeabilized with Cytofix/Cytoperm (BD BioSciences, catalog 554722). A 1× Perm Wash buffer was used to dilute intracellular antibodies. To measure intracellular markers, antibodies were used at a 1:100 dilution of p24 FITC (clone KC57, Beckman Coulter, cat# 6604665). Cells were incubated for 30 min at 4°C, washed using Perm Wash buffer, and then resuspended in FACS buffer until ready to be run on flow cytometer.

We calculated specific killing as a ratio of the percent of infected cells in the co-culture compared to the media control using the following formula:


$$Specific\;killing=\left(1-\frac{NK\;and\;CD4\;co-culture}{CD4\;culture\;media\;control}\right)\times 100$$

### MHC-I expression

Similar procedures of activating, expanding, and infecting naïve CD4 T cells were followed, as described above, in co-culture experiments. However, on day 12, the cells were uncrowded, counted, and resuspended at 1 × 10^6^ cells/mL in the presence of ART (raltegravir 1 μM and nelfinavir 0.5 μM), with the addition of the three retinoids (alitretinoin, tazarotene acid, and AM80) at a 1 μM concentration, with or without 100 ng/mL of IL-15. Cells were incubated at 37°C for 24 h and then stained for flow cytometry analysis. Cells were stained with a 1:100 dilution of antibodies with FACS buffer. Specifically, cells were stained with viability dye with 100 μL of FACS and then incubated at 4°C for 10 min. After washing the cells with FACS buffer, they were stained with anti-human CD4 and anti-human HLA-ABC APC (clone W6/32, BioLegend, cat# 311410), anti-human HLA-E PE (clone 3D12, BioLegend, cat# 342604), or anti-human HLA-F APC (clone 3D11, BioLegend, cat# 373208). After a 30-minute incubation at 4°C, cells were washed with FACS buffer, fixed, and permeabilized with Cytofix/Cytoperm, and then intracellularly stained for p24 as previously described in the staining procedure for co-culture experiments.

### NK phenotype

NK cells were isolated from PBMCs using the EasySep Human NK Cell Isolation Kit (Stem Cell, cat# 17955) and rested overnight. Cells were plated in the absence or presence of either alitretinoin, tazarotene acid, or AM80 at a 1 μM concentration with or without 100 ng/mL of IL-15. Cells were incubated at 37°C for 24 h and then stained. Cells were stained with a 1:100 dilution of antibodies in FACS buffer. Specifically, cells were stained with viability dye and 100 μL of FACS, then incubated at 4°C for 10 min. After washing the cells with FACS buffer, Human FC Block with 100 μL of FACS was added, and the cells were incubated at room temperature for 10 min. Following this, cells were stained extracellularly with anti-human CD3 BV786 (clone SP34-2, BD BioSciences, cat# 563918), anti-human CD56 BV605 (clone HCD56, BioLegend, cat# 318334), and anti-human CD16 FITC (clone 3G8, BD BioSciences, cat# 555406). For additional extracellular marker experiments, the cells were also stained with anti-human NKG2A BV421 (clone 131411, BD BioSciences, cat# 747924), anti-human NKG2C BV711 (clone 134591, BD BioSciences, cat# 748164), anti-human NKG2D PE-Dazzle 594 (clone 1D11, BioLegend, cat# 320828), recombinant human KIR3DS1/L1 PE (clone Z27.3.7, Beckman Coulter, cat# IM3292), and/or anti-human KIR3DL1 BV421 (clone DX9, BioLegend, cat# 312714). If only extracellular markers were being analyzed, cells were incubated at 4°C for 30 min then washed with FACS buffer and fixed with 3% PFA. If intracellular markers were being analyzed, the cells were incubated at 4°C for 30 min, washed, and then fixed and permeabilized with Cytofix/Cytoperm. After this, the cells were stained with anti-human granzyme A PE/Cy7 (clone CB9, BioLegend, cat# 507221), anti-human granzyme B Alexa Fluor 700 (clone GB11, BD BioSciences, cat# 56213), and/or anti-human Perforin FITC (clone dG9, BioLegend, cat# 308104).

All experiments were performed on either a BD LSR Fortessa X20 flow cytometer or a BD Celesta Analyzer with FACSDiva software. Data were analyzed using FlowJo (BD BioSciences, USA).

### Statistics

Statistical analyses were performed using GraphPad Prism. We used the Shapiro-Wilk statistical tests of normality to determine if the results were normally distributed. If the results passed this test, we assumed Gaussian distribution and analyzed data accordingly. If the results did not pass this test, we used non-parametric analyses. All statistical tests are indicated in the figure legends. All *P* values less than 0.05 were considered significant.

### Study approval

White blood cell concentrates (buffy coat), prepared from a single unit of whole blood by centrifugation, were purchased from Gulf Coast Regional Blood Center. Volunteers aged 17 years and older served as blood participants. No other personal information, besides age and biological sex, is provided.

## Data Availability

Values for data points shown in graphs are provided in the supplemental Source Data File. All additional data are provided in the supplemental files.

## AFTER EPUB

*[This article was published on 27 January 2026 with Jonathan S. Lochner's name misspelled. The error was corrected in the current version, posted on 28 January 2026.]*

## SUPPLEMENTAL MATERIAL

Supplemental figures jvi.01620-25-s0001.pdf Figures S1 to S12.

Supplemental source data file jvi.01620-25-s0002.xlsx Source data for all figures.

ASM does not own the copyrights to Supplemental Material that may be linked to, or accessed through, an article. The authors have granted ASM a non-exclusive, world-wide license to publish the Supplemental Material files. Please contact the corresponding author directly for reuse.

## References

[1] Chun TW, Stuyver L, Mizell SB, Ehler LA, Mican JA, Baseler M, Lloyd AL, Nowak MA, Fauci AS. 1997. Presence of an inducible HIV-1 latent reservoir during highly active antiretroviral therapy. Proc Natl Acad Sci USA 94:13193–13197. doi:10.1073/pnas.94.24.13193 9371822 PMC24285

[2] Finzi D, Hermankova M, Pierson T, Carruth LM, Buck C, Chaisson RE, Quinn TC, Chadwick K, Margolick J, Brookmeyer R, Gallant J, Markowitz M, Ho DD, Richman DD, Siliciano RF. 1997. Identification of a reservoir for HIV-1 in patients on highly active antiretroviral therapy. Science 278:1295–1300. doi:10.1126/science.278.5341.1295 9360927

[3] Siliciano JD, Kajdas J, Finzi D, Quinn TC, Chadwick K, Margolick JB, Kovacs C, Gange SJ, Siliciano RF. 2003. Long-term follow-up studies confirm the stability of the latent reservoir for HIV-1 in resting CD4+ T cells. Nat Med 9:727–728. doi:10.1038/nm880 12754504

[4] Terai C, Kornbluth RS, Pauza CD, Richman DD, Carson DA. 1991. Apoptosis as a mechanism of cell death in cultured T lymphoblasts acutely infected with HIV-1. J Clin Invest 87:1710–1715. doi:10.1172/JCI115188 2022741 PMC295273

[5] Miedema F, Meyaard L, Koot M, Klein MR, Roos MT, Groenink M, Fouchier RA, Van’t Wout AB, Tersmette M, Schellekens PT. 1994. Changing virus-host interactions in the course of HIV-1 infection. Immunol Rev 140:35–72. doi:10.1111/j.1600-065x.1994.tb00864.x 7821927

[6] Costin JM. 2007. Cytopathic mechanisms of HIV-1. Virol J 4:100. doi:10.1186/1743-422X-4-100 17945027 PMC2174939

[7] Wu G, Swanson M, Talla A, Graham D, Strizki J, Gorman D, Barnard RJ, Blair W, Søgaard OS, Tolstrup M, Østergaard L, Rasmussen TA, Sekaly RP, Archin NM, Margolis DM, Hazuda DJ, Howell BJ. 2017. HDAC inhibition induces HIV-1 protein and enables immune-based clearance following latency reversal. JCI Insight 2:e92901. doi:10.1172/jci.insight.92901 28814661 PMC5621903

[8] Jones RB, Mueller S, O’Connor R, Rimpel K, Sloan DD, Karel D, Wong HC, Jeng EK, Thomas AS, Whitney JB, et al.. 2016. A subset of latency-reversing agents expose HIV-infected resting CD4+ T-cells to recognition by cytotoxic T-lymphocytes. PLoS Pathog 12:e1005545. doi:10.1371/journal.ppat.1005545 27082643 PMC4833318

[9] Scully E, Alter G. 2016. NK cells in HIV disease. Curr HIV/AIDS Rep 13:85–94. doi:10.1007/s11904-016-0310-3 27002078 PMC4821863

[10] Mikulak J, Oriolo F, Zaghi E, Di Vito C, Mavilio D. 2017. Natural killer cells in HIV-1 infection and therapy. AIDS 31:2317–2330. doi:10.1097/QAD.0000000000001645 28926399 PMC5892189

[11] Alter G, Altfeld M. 2009. NK cells in HIV-1 infection: evidence for their role in the control of HIV-1 infection. J Intern Med 265:29–42. doi:10.1111/j.1365-2796.2008.02045.x 19093958 PMC2842208

[12] Alter Galit, Martin MP, Teigen N, Carr WH, Suscovich TJ, Schneidewind A, Streeck H, Waring M, Meier A, Brander C, Lifson JD, Allen TM, Carrington M, Altfeld M. 2007. Differential natural killer cell-mediated inhibition of HIV-1 replication based on distinct KIR/HLA subtypes. J Exp Med 204:3027–3036. doi:10.1084/jem.20070695 18025129 PMC2118524

[13] Hölzemer A, Garcia-Beltran WF, Altfeld M. 2017. Natural killer cell interactions with classical and non-classical human leukocyte antigen class I in HIV-1 infection. Front Immunol 8:1496. doi:10.3389/fimmu.2017.01496 29184550 PMC5694438

[14] Mavilio D, Benjamin J, Daucher M, Lombardo G, Kottilil S, Planta MA, Marcenaro E, Bottino C, Moretta L, Moretta A, Fauci AS. 2003. Natural killer cells in HIV-1 infection: dichotomous effects of viremia on inhibitory and activating receptors and their functional correlates. Proc Natl Acad Sci USA 100:15011–15016. doi:10.1073/pnas.2336091100 14645713 PMC299884

[15] Mavilio D, Lombardo G, Benjamin J, Kim D, Follman D, Marcenaro E, O’Shea MA, Kinter A, Kovacs C, Moretta A, Fauci AS. 2005. Characterization of CD56-/CD16+ natural killer (NK) cells: a highly dysfunctional NK subset expanded in HIV-infected viremic individuals. Proc Natl Acad Sci USA 102:2886–2891. doi:10.1073/pnas.0409872102 15699323 PMC549494

[16] Alter G, Malenfant JM, Delabre RM, Burgett NC, Yu XG, Lichterfeld M, Zaunders J, Altfeld M. 2004. Increased natural killer cell activity in viremic HIV-1 infection. J Immunol 173:5305–5311. doi:10.4049/jimmunol.173.8.5305 15470077

[17] Alter Galit, Teigen N, Davis BT, Addo MM, Suscovich TJ, Waring MT, Streeck H, Johnston MN, Staller KD, Zaman MT, Yu XG, Lichterfeld M, Basgoz N, Rosenberg ES, Altfeld M. 2005. Sequential deregulation of NK cell subset distribution and function starting in acute HIV-1 infection. Blood 106:3366–3369. doi:10.1182/blood-2005-03-1100 16002429

[18] Leeansyah E, Zhou J, Paukovics G, Lewin SR, Crowe SM, Jaworowski A. 2010. Decreased NK cell FcRγ in HIV-1 infected individuals receiving combination antiretroviral therapy: a cross sectional study. PLoS One 5:e9643. doi:10.1371/journal.pone.0009643 20224795 PMC2835768

[19] Lichtfuss GF, Cheng W-J, Farsakoglu Y, Paukovics G, Rajasuriar R, Velayudham P, Kramski M, Hearps AC, Cameron PU, Lewin SR, Crowe SM, Jaworowski A. 2012. Virologically suppressed HIV patients show activation of NK cells and persistent innate immune activation. J Immunol 189:1491–1499. doi:10.4049/jimmunol.1200458 22745371

[20] Berger C, Berger M, Hackman RC, Gough M, Elliott C, Jensen MC, Riddell SR. 2009. Safety and immunologic effects of IL-15 administration in nonhuman primates. Blood 114:2417–2426. doi:10.1182/blood-2008-12-189266 19605850 PMC2746471

[21] Conlon KC, Lugli E, Welles HC, Rosenberg SA, Fojo AT, Morris JC, Fleisher TA, Dubois SP, Perera LP, Stewart DM, Goldman CK, Bryant BR, Decker JM, Chen J, Worthy TA, Figg WD Sr, Peer CJ, Sneller MC, Lane HC, Yovandich JL, Creekmore SP, Roederer M, Waldmann TA. 2015. Redistribution, hyperproliferation, activation of natural killer cells and CD8 T cells, and cytokine production during first-in-human clinical trial of recombinant human interleukin-15 in patients with cancer. J Clin Oncol 33:74–82. doi:10.1200/JCO.2014.57.3329 25403209 PMC4268254

[22] Seay K, Church C, Zheng JH, Deneroff K, Ochsenbauer C, Kappes JC, Liu B, Jeng EK, Wong HC, Goldstein H. 2015. In vivo activation of human NK cells by treatment with an interleukin-15 superagonist potently inhibits acute in vivo HIV-1 infection in humanized mice. J Virol 89:6264–6274. doi:10.1128/JVI.00563-15 25833053 PMC4474292

[23] Chehimi J, Marshall JD, Salvucci O, Frank I, Chehimi S, Kawecki S, Bacheller D, Rifat S, Chouaib S. 1997. IL-15 enhances immune functions during HIV infection. J Immunol 158:5978–5987. doi:10.4049/jimmunol.158.12.5978 9190952

[24] Covino DA, Desimio MG, Doria M. 2022. Impact of IL-15 and latency reversing agent combinations in the reactivation and NK cell-mediated suppression of the HIV reservoir. Sci Rep 12:18567. doi:10.1038/s41598-022-23010-5 36329160 PMC9633760

[25] Romee R, Cooley S, Berrien-Elliott MM, Westervelt P, Verneris MR, Wagner JE, Weisdorf DJ, Blazar BR, Ustun C, DeFor TE, et al.. 2018. First-in-human phase 1 clinical study of the IL-15 superagonist complex ALT-803 to treat relapse after transplantation. Blood 131:2515–2527. doi:10.1182/blood-2017-12-823757 29463563 PMC5992862

[26] Miller JS, Davis ZB, Helgeson E, Reilly C, Thorkelson A, Anderson J, Lima NS, Jorstad S, Hart GT, Lee JH, Safrit JT, Wong H, Cooley S, Gharu L, Chung H, Soon-Shiong P, Dobrowolski C, Fletcher CV, Karn J, Douek DC, Schacker TW. 2022. Safety and virologic impact of the IL-15 superagonist N-803 in people living with HIV: a phase 1 trial. Nat Med 28:392–400. doi:10.1038/s41591-021-01651-9 35102335

[27] Macedo AB, Levinger C, Nguyen BN, Richard J, Gupta M, Cruz CRY, Finzi A, Chiappinelli KB, Crandall KA, Bosque A. 2022. The HIV latency reversal agent HODHBt enhances NK cell effector and memory-like functions by increasing interleukin-15-mediated STAT activation. J Virol 96:e0037222. doi:10.1128/jvi.00372-22 35867565 PMC9364794

[28] Garrido C, Abad-Fernandez M, Tuyishime M, Pollara JJ, Ferrari G, Soriano-Sarabia N, Margolis DM. 2018. Interleukin-15-stimulated natural killer cells clear HIV-1-infected cells following latency reversal ex vivo. J Virol 92:00235–18. doi:10.1128/JVI.00235-18 PMC5974478 29593039

[29] Perera PY, Lichy JH, Waldmann TA, Perera LP. 2012. The role of interleukin-15 in inflammation and immune responses to infection: implications for its therapeutic use. Microbes Infect 14:247–261. doi:10.1016/j.micinf.2011.10.006 22064066 PMC3270128

[30] Chang KH, Kim JM, Kim HY, Song YG, Choi YH, Park YS, Cho JH, Hong SK. 2000. Spontaneous programmed cell death of peripheral blood mononuclear cells from HIV-infected persons is decreased with interleukin-15. Yonsei Med J 41:112. doi:10.3349/ymj.2000.41.1.112 10731928

[31] Milush JM, López-Vergès S, York VA, Deeks SG, Martin JN, Hecht FM, Lanier LL, Nixon DF. 2013. CD56negCD16 + CD56negCD16. Retrovirology (Auckl) 10:158. doi:10.1186/1742-4690-10-158 PMC3892122 24351015

[32] Kramski M, Stratov I, Kent SJ. 2015. The role of HIV-specific antibody-dependent cellular cytotoxicity in HIV prevention and the influence of the HIV-1 Vpu protein. AIDS 29:137–144. doi:10.1097/QAD.0000000000000523 25396265

[33] Lee WS, Parsons MS, Kent SJ, Lichtfuss M. 2015. Can HIV-1-specific ADCC assist the clearance of reactivated latently infected cells? Front Immunol 6:265. doi:10.3389/fimmu.2015.00265 26074924 PMC4445400

[34] McCoy LE. 2018. The expanding array of HIV broadly neutralizing antibodies. Retrovirology (Auckl) 15:70. doi:10.1186/s12977-018-0453-y PMC6192334 30326938

[35] Alvarez RA, Hamlin RE, Monroe A, Moldt B, Hotta MT, Rodriguez Caprio G, Fierer DS, Simon V, Chen BK. 2014. HIV-1 Vpu antagonism of tetherin inhibits antibody-dependent cellular cytotoxic responses by natural killer cells. J Virol 88:6031–6046. doi:10.1128/JVI.00449-14 24623433 PMC4093850

[36] Pham TN, Lukhele S, Hajjar F, Routy J-P, Cohen ÉA. 2014. HIV Nef and Vpu protect HIV-infected CD4+ T cells from antibody-mediated cell lysis through down-modulation of CD4 and BST2. Retrovirology (Auckl) 11:15. doi:10.1186/1742-4690-11-15 PMC3930549 24498878

[37] Barouch DH, Whitney JB, Moldt B, Klein F, Oliveira TY, Liu J, Stephenson KE, Chang H-W, Shekhar K, Gupta S, et al.. 2013. Therapeutic efficacy of potent neutralizing HIV-1-specific monoclonal antibodies in SHIV-infected rhesus monkeys. Nature 503:224–228. doi:10.1038/nature12744 24172905 PMC4017780

[38] Richard J, Veillette M, Brassard N, Iyer SS, Roger M, Martin L, Pazgier M, Schön A, Freire E, Routy J-P, Smith AB 3rd, Park J, Jones DM, Courter JR, Melillo BN, Kaufmann DE, Hahn BH, Permar SR, Haynes BF, Madani N, Sodroski JG, Finzi A. 2015. CD4 mimetics sensitize HIV-1-infected cells to ADCC. Proc Natl Acad Sci USA 112:E2687–94. doi:10.1073/pnas.1506755112 25941367 PMC4443331

[39] Chung AW, Isitman G, Navis M, Kramski M, Center RJ, Kent SJ, Stratov I. 2011. Immune escape from HIV-specific antibody-dependent cellular cytotoxicity (ADCC) pressure. Proc Natl Acad Sci USA 108:7505–7510. doi:10.1073/pnas.1016048108 21502492 PMC3088575

[40] Bruel T, Guivel-Benhassine F, Amraoui S, Malbec M, Richard L, Bourdic K, Donahue DA, Lorin V, Casartelli N, Noël N, Lambotte O, Mouquet H, Schwartz O. 2016. Elimination of HIV-1-infected cells by broadly neutralizing antibodies. Nat Commun 7:10844. doi:10.1038/ncomms10844 26936020 PMC4782064

[41] Halper-Stromberg A, Lu CL, Klein F, Horwitz JA, Bournazos S, Nogueira L, Eisenreich TR, Liu C, Gazumyan A, Schaefer U, Furze RC, Seaman MS, Prinjha R, Tarakhovsky A, Ravetch JV, Nussenzweig MC. 2014. Broadly neutralizing antibodies and viral inducers decrease rebound from HIV-1 latent reservoirs in humanized mice. Cell 158:989–999. doi:10.1016/j.cell.2014.07.043 25131989 PMC4163911

[42] Klein F, Nogueira L, Nishimura Y, Phad G, West AP, Halper-Stromberg A, Horwitz JA, Gazumyan A, Liu C, Eisenreich TR, Lehmann C, Fätkenheuer G, Williams C, Shingai M, Martin MA, Bjorkman PJ, Seaman MS, Zolla-Pazner S, Karlsson Hedestam GB, Nussenzweig MC. 2014. Enhanced HIV-1 immunotherapy by commonly arising antibodies that target virus escape variants. J Exp Med 211:2361–2372. doi:10.1084/jem.20141050 25385756 PMC4235636

[43] Garcia-Vidal E, Castellví M, Pujantell M, Badia R, Jou A, Gomez L, Puig T, Clotet B, Ballana E, Riveira-Muñoz E, Esté JA. 2017. Evaluation of the innate immune modulator acitretin as a strategy to clear the HIV reservoir. Antimicrob Agents Chemother 61:01368–17. doi:10.1128/AAC.01368-17 PMC5655051 28874382

[44] Purssell A, McGuinty M, Vulesevic B, Angel JB. 2022. Retinoids: novel potential therapeutics in the pursuit of HIV-1 cure. Front Virol 2:957124. doi:10.3389/fviro.2022.957124

[45] Li P, Kaiser P, Lampiris HW, Kim P, Yukl SA, Havlir DV, Greene WC, Wong JK. 2016. Stimulating the RIG-I pathway to kill cells in the latent HIV reservoir following viral reactivation. Nat Med 22:807–811. doi:10.1038/nm.4124 27294875 PMC5004598

[46] Howard JN, Levinger C, Deletsu S, Fromentin R, Chomont N, Bosque A, for the AIDS Clinical Trials Group (ACTG) A5325 Team. 2024. Isotretinoin promotes elimination of translation-competent HIV latent reservoirs in CD4T cells. PLoS Pathog 20:e1012601. doi:10.1371/journal.ppat.1012601 39401241 PMC11501018

[47] Altucci L, Gronemeyer H. 2001. The promise of retinoids to fight against cancer. Nat Rev Cancer 1:181–193. doi:10.1038/35106036 11902573

[48] Muindi JR, Frankel SR, Huselton C, DeGrazia F, Garland WA, Young CW, Warrell RP Jr. 1992. Clinical pharmacology of oral all-trans retinoic acid in patients with acute promyelocytic leukemia. Cancer Res 52:2138–2142. 1559217

[49] Sporn MB, Roberts AB. 1984. Role of retinoids in differentiation and carcinogenesis. J Natl Cancer Inst 73:1381–1387. 6595447

[50] Dogra S, Jain A, Kanwar AJ. 2013. Efficacy and safety of acitretin in three fixed doses of 25, 35 and 50 mg in adult patients with severe plaque type psoriasis: a randomized, double blind, parallel group, dose ranging study. Acad Dermatol Venereol 27:e305–e311. doi:10.1111/j.1468-3083.2012.04644.x 22816881

[51] Nakamura M, Abrouk M, Farahnik B, Zhu TH, Bhutani T. 2018. Psoriasis treatment in HIV-positive patients: a systematic review of systemic immunosuppressive therapies. Cutis 101:38. 29529104

[52] BALL S, Goodwin TW, MORTON RA. 1946. Retinene1-vitamin A aldehyde. Biochem J 40:lix. 20341217

[53] Wald G. 1948. The synthesis from vitamin A1 of “retinene1” and of a new 545 mµ chromogen yielding light-sensitive products. J Gen Physiol 31:489–504. doi:10.1085/jgp.31.6.489 18870869 PMC2147125

[54] Chomienne C, Ballerini P, Balitrand N, Huang ME, Krawice I, Castaigne S, Fenaux P, Tiollais P, Dejean A, Degos L, de The H. 1990. The retinoic acid receptor alpha gene is rearranged in retinoic acid-sensitive promyelocytic leukemias. Leukemia 4:802–807. 2173802

[55] Kang S. 1998. Photoaging and tretinoin. Dermatol Clin 16:357–364. doi:10.1016/s0733-8635(05)70018-8 9589209

[56] Tsukada M, Schröder M, Roos TC, Chandraratna RA, Reichert U, Merk HF, Orfanos CE, Zouboulis CC. 2000. 13-cis retinoic acid exerts its specific activity on human sebocytes through selective intracellular isomerization to all-trans retinoic acid and binding to retinoid acid receptors. J Invest Dermatol 115:321–327. doi:10.1046/j.1523-1747.2000.00066.x 10951254

[57] Lotan R, Xu XC, Lippman SM, Ro JY, Lee JS, Lee JJ, Hong WK. 1995. Suppression of retinoic acid receptor-beta in premalignant oral lesions and its up-regulation by isotretinoin. N Engl J Med 332:1405–1410. doi:10.1056/NEJM199505253322103 7723796

[58] Cheng C, Michaels J, Scheinfeld N. 2008. Alitretinoin: a comprehensive review. Expert Opin Investig Drugs 17:437–443. doi:10.1517/13543784.17.3.437 18321241

[59] Allenby G, Bocquel MT, Saunders M, Kazmer S, Speck J, Rosenberger M, Lovey A, Kastner P, Grippo JF, Chambon P. 1993. Retinoic acid receptors and retinoid X receptors: interactions with endogenous retinoic acids. Proc Natl Acad Sci USA 90:30–34. doi:10.1073/pnas.90.1.30 8380496 PMC45593

[60] Siegenthaler G, Saurat JH. 1986. Therapy with a synthetic retinoid--(Ro 10-1670) etretin--increases the cellular retinoic acid-binding protein in nonlesional psoriatic skin. J Invest Dermatol 87:122–124. doi:10.1111/1523-1747.ep12523628 2425004

[61] LeMotte PK, Keidel S, Apfel CM. 1996. Characterization of synthetic retinoids with selectivity for retinoic acid or retinoid X nuclear receptors. Biochim Biophys Acta 1289:298–304. doi:10.1016/0304-4165(95)00179-4 8600988

[62] Bernard BA. 1993. Adapalene, a new chemical entity with retinoid activity. Skin Pharmacol 6 Suppl 1:61–69. doi:10.1159/000211165 8142113

[63] Shroot B, Michel S. 1997. Pharmacology and chemistry of adapalene. J Am Acad Dermatol 36:S96–103. doi:10.1016/s0190-9622(97)70050-1 9204085

[64] Boehm MF, Zhang L, Zhi L, McClurg MR, Berger E, Wagoner M, Mais DE, Suto CM, Davies JA, Heyman RA. 1995. Design and synthesis of potent retinoid X receptor selective ligands that induce apoptosis in leukemia cells. J Med Chem 38:3146–3155. doi:10.1021/jm00016a018 7636877

[65] Chandraratna RA. 1996. Tazarotene--first of a new generation of receptor-selective retinoids. Br J Dermatol 135 Suppl 49:18–25. doi:10.1111/j.1365-2133.1996.tb15662.x 9035701

[66] Menter A. 2000. Pharmacokinetics and safety of tazarotene. J Am Acad Dermatol 43:S31–S35. doi:10.1067/mjd.2000.108321 10898827

[67] Nagpal S, Athanikar J, Chandraratna RA. 1995. Separation of transactivation and AP1 antagonism functions of retinoic acid receptor alpha. J Biol Chem 270:923–927. doi:10.1074/jbc.270.2.923 7822331

[68] Miwako I, Kagechika H. 2007. Tamibarotene. Drugs Today (Barc) 43:563–568. doi:10.1358/dot.2007.43.8.1072615 17925887

[69] Delescluse C, Cavey MT, Martin B, Bernard BA, Reichert U, Maignan J, Darmon M, Shroot B. 1991. Selective high affinity retinoic acid receptor alpha or beta-gamma ligands. Mol Pharmacol 40:556–562. 1656191

[70] Hind M, Stinchcombe S. 2009. Palovarotene, a novel retinoic acid receptor gamma agonist for the treatment of emphysema. Curr Opin Investig Drugs 10:1243–1250. 19876792

[71] Helleboid S, Haug C, Lamottke K, Zhou Y, Wei J, Daix S, Cambula L, Rigou G, Hum DW, Walczak R. 2014. The identification of naturally occurring neoruscogenin as a bioavailable, potent, and high-affinity agonist of the nuclear receptor RORα (NR1F1). J Biomol Screen 19:399–406. doi:10.1177/1087057113497095 23896689

[72] Chang MR, Dharmarajan V, Doebelin C, Garcia-Ordonez RD, Novick SJ, Kuruvilla DS, Kamenecka TM, Griffin PR. 2016. Synthetic RORγt agonists enhance protective immunity. ACS Chem Biol 11:1012–1018. doi:10.1021/acschembio.5b00899 26785144 PMC5178133

[73] Bliss CI. 1939. The toxicity of poisons applied jointly1. Ann Appl Biol 26:585–615. doi:10.1111/j.1744-7348.1939.tb06990.x

[74] Baalwa J, Wang S, Parrish NF, Decker JM, Keele BF, Learn GH, Yue L, Ruzagira E, Ssemwanga D, Kamali A, Amornkul PN, Price MA, Kappes JC, Karita E, Kaleebu P, Sanders E, Gilmour J, Allen S, Hunter E, Montefiori DC, Haynes BF, Cormier E, Hahn BH, Shaw GM. 2013. Molecular identification, cloning and characterization of transmitted/founder HIV-1 subtype A, D and A/D infectious molecular clones. Virology (Auckl) 436:33–48. doi:10.1016/j.virol.2012.10.009 PMC3545109 23123038

[75] Prager I, Watzl C. 2019. Mechanisms of natural killer cell-mediated cellular cytotoxicity. J Leukoc Biol 105:1319–1329. doi:10.1002/JLB.MR0718-269R 31107565

[76] Guo Y, Luan L, Rabacal W, Bohannon JK, Fensterheim BA, Hernandez A, Sherwood ER. 2015. IL-15 superagonist-mediated immunotoxicity: role of NK cells and IFN-γ. J Immunol 195:2353–2364. doi:10.4049/jimmunol.1500300 26216888 PMC4543906

[77] Lin SJ, Kuo ML, Hsiao HS, Lee PT, Lee WI, Chen JY, Huang JL. 2019. Cytotoxic function and cytokine production of natural killer cells and natural killer T-like cells in systemic lupus erythematosis regulation with interleukin-15. Mediators Inflamm 2019:1–12. doi:10.1155/2019/4236562 PMC6462338 31049024

[78] Aktas E, Kucuksezer UC, Bilgic S, Erten G, Deniz G. 2009. Relationship between CD107a expression and cytotoxic activity. Cell Immunol 254:149–154. doi:10.1016/j.cellimm.2008.08.007 18835598

[79] Bryceson YT, March ME, Ljunggren HG, Long EO. 2006. Activation, coactivation, and costimulation of resting human natural killer cells. Immunol Rev 214:73–91. doi:10.1111/j.1600-065X.2006.00457.x 17100877 PMC3845883

[80] Frankel AD, Young JA. 1998. HIV-1: fifteen proteins and an RNA. Annu Rev Biochem 67:1–25. doi:10.1146/annurev.biochem.67.1.1 9759480

[81] Lindwasser OW, Chaudhuri R, Bonifacino JS. 2007. Mechanisms of CD4 downregulation by the Nef and Vpu proteins of primate immunodeficiency viruses. Curr Mol Med 7:171–184. doi:10.2174/156652407780059177 17346169

[82] Garcia-Beltran WF, Hölzemer A, Martrus G, Chung AW, Pacheco Y, Simoneau CR, Rucevic M, Lamothe-Molina PA, Pertel T, Kim T-E, Dugan H, Alter G, Dechanet-Merville J, Jost S, Carrington M, Altfeld M. 2016. Open conformers of HLA-F are high-affinity ligands of the activating NK-cell receptor KIR3DS1. Nat Immunol 17:1067–1074. doi:10.1038/ni.3513 27455421 PMC4992421

[83] Kiani Z, Bruneau J, Geraghty DE, Bernard NF. 2019. HLA-F on autologous HIV-infected cells activates primary NK cells expressing the activating killer immunoglobulin-like receptor KIR3DS1. J Virol 93:00933–19. doi:10.1128/JVI.00933-19 PMC6714807 31270222

[84] Burian A, Wang KL, Finton KAK, Lee N, Ishitani A, Strong RK, Geraghty DE. 2016. HLA-F and MHC-I open conformers bind natural killer cell Ig-like receptor KIR3DS1. PLoS One 11:e0163297. doi:10.1371/journal.pone.0163297 27649529 PMC5029895

[85] Pascal V, Yamada E, Martin MP, Alter G, Altfeld M, Metcalf JA, Baseler MW, Adelsberger JW, Carrington M, Anderson SK, McVicar DW. 2007. Detection of KIR3DS1 on the cell surface of peripheral blood NK cells facilitates identification of a novel null allele and assessment of KIR3DS1 expression during HIV-1 Infection. The Journal of Immunology 179:1625–1633. doi:10.4049/jimmunol.179.3.1625 17641029

[86] Trundley A, Frebel H, Jones D, Chang C, Trowsdale J. 2007. Allelic expression patterns of KIR3DS1 and 3DL1 using the Z27 and DX9 antibodies. Eur J Immunol 37:780–787. doi:10.1002/eji.200636773 17301953

[87] Parham P, Norman PJ, Abi-Rached L, Guethlein LA. 2011. Variable NK Cell Receptors Exemplified by Human KIR3DL1/S1. J Immunol 187:11–19. doi:10.4049/jimmunol.0902332 21690332 PMC3223120

[88] Yawata M, Yawata N, Draghi M, Little AM, Partheniou F, Parham P. 2006. Roles for HLA and KIR polymorphisms in natural killer cell repertoire selection and modulation of effector function. J Exp Med 203:633–645. doi:10.1084/jem.20051884 16533882 PMC2118260

[89] Boudreau JE, Le Luduec JB, Hsu KC. 2014. Development of a novel multiplex PCR assay to detect functional subtypes of KIR3DL1 alleles. PLoS One 9:e99543. doi:10.1371/journal.pone.0099543 24919192 PMC4053526

[90] Gardiner CM, Guethlein LA, Shilling HG, Pando M, Carr WH, Rajalingam R, Vilches C, Parham P. 2001. Different NK cell surface phenotypes defined by the DX9 antibody are due to KIR3DL1 gene polymorphism. J Immunol 166:2992–3001. doi:10.4049/jimmunol.166.5.2992 11207248

[91] Morvan M, Willem C, Gagne K, Kerdudou N, David G, Sébille V, Folléa G, Bignon JD, Retière C. 2009. Phenotypic and functional analyses of KIR3DL1+ and KIR3DS1+ NK cell subsets demonstrate differential regulation by Bw4 molecules and induced KIR3DS1 expression on stimulated NK cells. J Immunol 182:6727–6735. doi:10.4049/jimmunol.0900212 19454667

[92] Jabea Ekabe C, Asaba Clinton N, Agyei EK, Kehbila J. 2022. Role of apoptosis in HIV pathogenesis. Adv Virol 2022:1–10. doi:10.1155/2022/8148119 PMC9023228 35462964

[93] Kapser C, Herzinger T, Ruzicka T, Flaig M, Molin S. 2015. Treatment of cutaneous T‐cell lymphoma with oral alitretinoin. Acad Dermatol Venereol 29:783–788. doi:10.1111/jdv.12684 25175592

[94] Kaemmerer T, Stadler PC, Helene Frommherz L, Guertler A, Einar French L, Reinholz M. 2021. Alitretinoin in the treatment of cutaneous T‐cell lymphoma. Cancer Med 10:7071–7078. doi:10.1002/cam4.4237 34435474 PMC8525105

[95] Napolitano M, Potestio L, De Lucia M, Nocerino M, Fabbrocini G, Patruno C. 2022. Alitretinoin for the treatment of severe chronic eczema of the hands. Expert Opin Pharmacother 23:159–167. doi:10.1080/14656566.2021.1998457 34789049

[96] Han G, Wu JJ, Del Rosso JQ. 2020. Use of topical tazarotene for the treatment of acne vulgaris in pregnancy: a literature review. J Clin Aesthet Dermatol 13:E59–E65. PMC7577328 33133344

[97] Venkataswamy P, Samudrala Venkatesiah S, Rao RS, Banavar SR, Patil S, Augustine D, Haragannavar VC. 2021. Immunohistochemical expression of tazarotene‐induced Gene 3 in oral squamous cell carcinoma. J Oral Pathology Medicine 50:403–409. doi:10.1111/jop.13144 33259689

[98] Hamzehpour H, Óskarsdóttir Á, Jónsson H, Jónsdóttir F, Sigurjónsson ÓE, Snorradottir BS. 2023. Transdermal drug delivery of tazarotene: determining tazarotene’s potential in local transdermal therapy. Pharmaceutics 16:64. doi:10.3390/pharmaceutics16010064 38258075 PMC10820539

[99] Dando TM, Wellington K. 2005. Topical tazarotene: a review of its use in the treatment of plaque psoriasis. Am J Clin Dermatol 6:255–272. doi:10.2165/00128071-200506040-00006 16060713

[100] Shinagawa K, Yanada M, Sakura T, Ueda Y, Sawa M, Miyatake J, Dobashi N, Kojima M, Hatta Y, Emi N, Tamaki S, Gomyo H, Yamazaki E, Fujimaki K, Asou N, Matsuo K, Ohtake S, Miyazaki Y, Ohnishi K, Kobayashi Y, Naoe T. 2014. Tamibarotene as maintenance therapy for acute promyelocytic leukemia: results from a randomized controlled trial. J Clin Oncol 32:3729–3735. doi:10.1200/JCO.2013.53.3570 25245439

[101] Tobita T, Takeshita A, Kitamura K, Ohnishi K, Yanagi M, Hiraoka A, Karasuno T, Takeuchi M, Miyawaki S, Ueda R, Naoe T, Ohno R. 1997. Treatment with a new synthetic retinoid, Am80, of acute promyelocytic leukemia relapsed from complete remission induced by all-trans retinoic acid. Blood 90:967–973. 9242525

[102] Alter G, Heckerman D, Schneidewind A, Fadda L, Kadie CM, Carlson JM, Oniangue-Ndza C, Martin M, Li B, Khakoo SI, Carrington M, Allen TM, Altfeld M. 2011. HIV-1 adaptation to NK-cell-mediated immune pressure. Nature 476:96–100. doi:10.1038/nature10237 21814282 PMC3194000

[103] Jost S, Altfeld M. 2012. Evasion from NK cell-mediated immune responses by HIV-1. Microbes Infect 14:904–915. doi:10.1016/j.micinf.2012.05.001 22626930 PMC3432664

[104] Kumar Mishra S, Kumar N, Or Rashid MH, Sultana S, Dawoud TM, Bourhia M, Georrge JJ. 2025. An integrated mutation-based immunoinformatic approach incorporating variability in epitopes: a study based on HIV subtype C. Front Immunol 16:1540253. doi:10.3389/fimmu.2025.1540253 40463364 PMC12129944

[105] Kiepiela P, Ngumbela K, Thobakgale C, Ramduth D, Honeyborne I, Moodley E, Reddy S, de Pierres C, Mncube Z, Mkhwanazi N, et al.. 2007. CD8+ T-cell responses to different HIV proteins have discordant associations with viral load. Nat Med 13:46–53. doi:10.1038/nm1520 17173051

[106] Fehniger TA, Caligiuri MA. 2001. Interleukin 15: biology and relevance to human disease. Blood 97:14–32. doi:10.1182/blood.v97.1.14 11133738

[107] Waldmann TA. 2013. The biology of IL-15: implications for cancer therapy and the treatment of autoimmune disorders. J Investig Dermatol Symp Proc 16:S28–30. doi:10.1038/jidsymp.2013.8 24326545

[108] Nattermann J, Nischalke HD, Hofmeister V, Kupfer B, Ahlenstiel G, Feldmann G, Rockstroh J, Weiss EH, Sauerbruch T, Spengler U. 2005. HIV-1 infection leads to increased HLA-E expression resulting in impaired function of natural killer cells. Antivir Ther (Lond) 10:95–107. doi:10.1177/135965350501000107 15751767

[109] Davis ZB, Cogswell A, Scott H, Mertsching A, Boucau J, Wambua D, Le Gall S, Planelles V, Campbell KS, Barker E. 2016. A conserved HIV-1-derived peptide presented by HLA-E renders infected T-cells highly susceptible to attack by NKG2A/CD94-bearing natural killer cells. PLoS Pathog 12:e1005421. doi:10.1371/journal.ppat.1005421 26828202 PMC4735451

[110] van Stigt Thans T, Akko JI, Niehrs A, Garcia-Beltran WF, Richert L, Stürzel CM, Ford CT, Li H, Ochsenbauer C, Kappes JC, Hahn BH, Kirchhoff F, Martrus G, Sauter D, Altfeld M, Hölzemer A. 2019. Primary HIV-1 strains use nef to downmodulate HLA-E surface expression. J Virol 93:e00719-19. doi:10.1128/JVI.00719-19 31375574 PMC6798123

[111] Romero-Martín L, Duran-Castells C, Olivella M, Rosás-Umbert M, Ruiz-Riol M, Sanchez J, Hartigan-O´Connor D, Mothe B, Olvera À, Brander C. 2022. Disruption of the HLA-E/NKG2X axis is associated with uncontrolled HIV infections. Front Immunol 13:1027855. doi:10.3389/fimmu.2022.1027855 36466823 PMC9716355

[112] Wallace Z, Heunis T, Paterson RL, Suckling RJ, Grant T, Dembek M, Donoso J, Brener J, Long J, Bunjobpol W, Gibbs-Howe D, Kay DP, Leneghan DB, Godinho LF, Walker A, Singh PK, Knox A, Leonard S, Dorrell L. 2024. Instability of the HLA-E peptidome of HIV presents a major barrier to therapeutic targeting. Mol Ther 32:678–688. doi:10.1016/j.ymthe.2024.01.010 38219014 PMC10928138

[113] Boyle LH, Gillingham AK, Munro S, Trowsdale J. 2006. Selective export of HLA-F by its cytoplasmic tail. J Immunol 176:6464–6472. doi:10.4049/jimmunol.176.11.6464 16709803

[114] Goodridge JP, Burian A, Lee N, Geraghty DE. 2013. HLA-F and MHC class i open conformers are ligands for NK cell ig-like receptors. J Immunol 191:3553–3562. doi:10.4049/jimmunol.1300081 24018270 PMC3780715

[115] Lin A, Yan WH. 2019. The emerging roles of human leukocyte antigen-F in immune modulation and viral infection. Front Immunol 10:964. doi:10.3389/fimmu.2019.00964 31134067 PMC6524545

[116] Körner C, Altfeld M. 2012. Role of KIR3DS1 in human diseases. Front Immunol 3:326. doi:10.3389/fimmu.2012.00326 23125843 PMC3485674

[117] Carr WH, Rosen DB, Arase H, Nixon DF, Michaelsson J, Lanier LL. 2007. Cutting edge: KIR3DS1, a gene implicated in resistance to progression to AIDS, encodes a DAP12-associated receptor expressed on NK cells that triggers NK cell activation. J Immunol 178:647–651. doi:10.4049/jimmunol.178.2.647 17202323 PMC2561215

[118] O’Connor GM, Guinan KJ, Cunningham RT, Middleton D, Parham P, Gardiner CM. 2007. Functional polymorphism of the KIR3DL1/S1 receptor on human NK cells. J Immunol 178:235–241. doi:10.4049/jimmunol.178.1.235 17182560

[119] Griffith SA, McCoy LE. 2021. To bnAb or not to bnAb: defining broadly neutralising antibodies against HIV-1. Front Immunol 12:708227. doi:10.3389/fimmu.2021.708227 34737737 PMC8560739

[120] LaMont C, Otwinowski J, Vanshylla K, Gruell H, Klein F, Nourmohammad A. 2022. Design of an optimal combination therapy with broadly neutralizing antibodies to suppress HIV-1. eLife 11:e76004. doi:10.7554/eLife.76004 35852143 PMC9467514

[121] Julg B, Barouch D. 2021. Broadly neutralizing antibodies for HIV-1 prevention and therapy. Semin Immunol 51:101475. doi:10.1016/j.smim.2021.101475 33858765

[122] Huang J, Kang BH, Ishida E, Zhou T, Griesman T, Sheng Z, Wu F, Doria-Rose NA, Zhang B, McKee K, et al.. 2016. Identification of a CD4-binding-site antibody to HIV that evolved near-pan neutralization breadth. Immunity 45:1108–1121. doi:10.1016/j.immuni.2016.10.027 27851912 PMC5770152

[123] Gómez-Mora E, Carrillo J, Urrea V, Rigau J, Alegre J, Cabrera C, Oltra E, Castro-Marrero J, Blanco J. 2020. Impact of long-term cryopreservation on blood immune cell markers in myalgic encephalomyelitis/chronic fatigue syndrome: implications for biomarker discovery. Front Immunol 11:582330. doi:10.3389/fimmu.2020.582330 33329554 PMC7732598

[124] Lauer FT, Denson JL, Burchiel SW. 2017. Isolation, cryopreservation, and immunophenotyping of human peripheral blood mononuclear cells. Curr Protoc Toxicol 74:18. doi:10.1002/cptx.31 PMC5726299 29117436

[125] Martin MP, Pascal V, Yeager M, Phair J, Kirk GD, Hoots K, O’Brien SJ, Anderson SK, Carrington M. 2007. A mutation in KIR3DS1 that results in truncation and lack of cell surface expression. Immunogenetics 59:823–829. doi:10.1007/s00251-007-0240-8 17687550

[126] Augusto DG, Norman PJ, Dandekar R, Hollenbach JA. 2019. Fluctuating and geographically specific selection characterize rapid evolution of the human KIR region. Front Immunol 10:989. doi:10.3389/fimmu.2019.00989 31156615 PMC6533848

[127] Boulet S, Sharafi S, Simic N, Bruneau J, Routy JP, Tsoukas CM, Bernard NF. 2008. Increased proportion of KIR3DS1 homozygotes in HIV-exposed uninfected individuals. AIDS 22:595–599. doi:10.1097/QAD.0b013e3282f56b23 18317000

[128] O’Connor GM, McVicar D. 2013. The yin-yang of KIR3DL1/S1: molecular mechanisms and cellular function. Crit Rev Immunol 33:203–218. doi:10.1615/critrevimmunol.2013007409 23756244 PMC3741655

