# Supplementary material for: Retinoids enhance NK effector function against HIV-infected CD4 T cells
Source: J Virol. 2026 Jan 27;100(2):e01620-25. doi: 10.1128/jvi.01620-25 (PMC12911896; doi:10.1128/jvi.01620-25)
Supplement: Supplemental figures — Figures S1 to S12. [file jvi.01620-25-s0001.pdf]

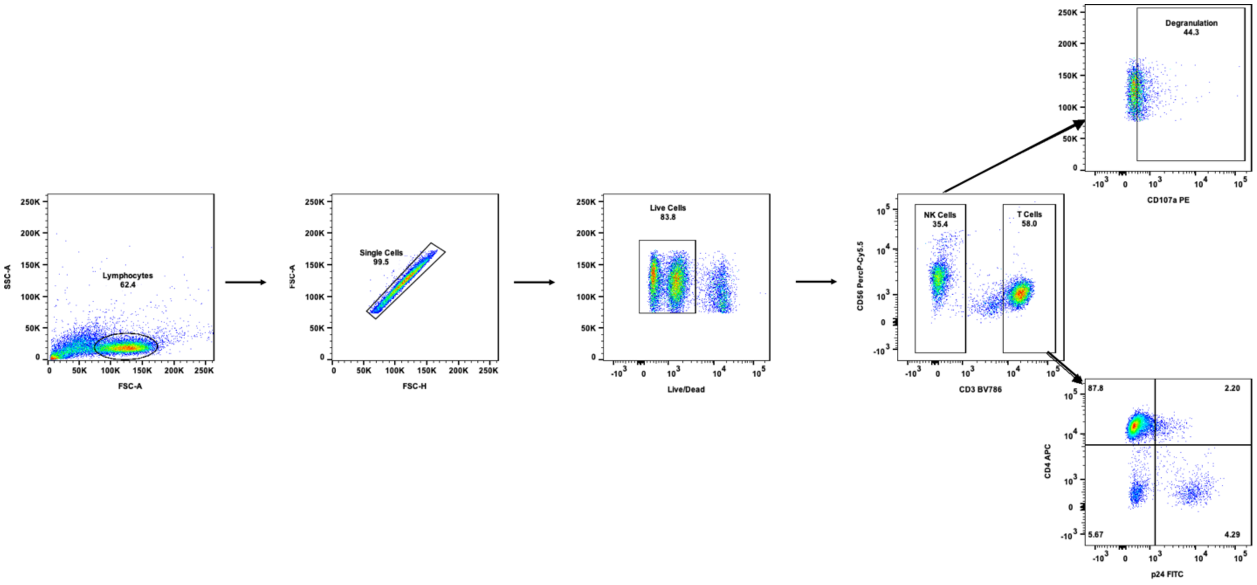

Supplemental Figure 1. **Co-culture gating strategy for Figure 1**. Cells were gated first based on lymphocytes followed by single cells then live cells. NK and CD4 T cells were separated based on the expression of CD56 and CD3. CD4 T cells were further gated out to determine uninfected and productively infected populations. The degranulation marker, CD107a was measured on NK cells.

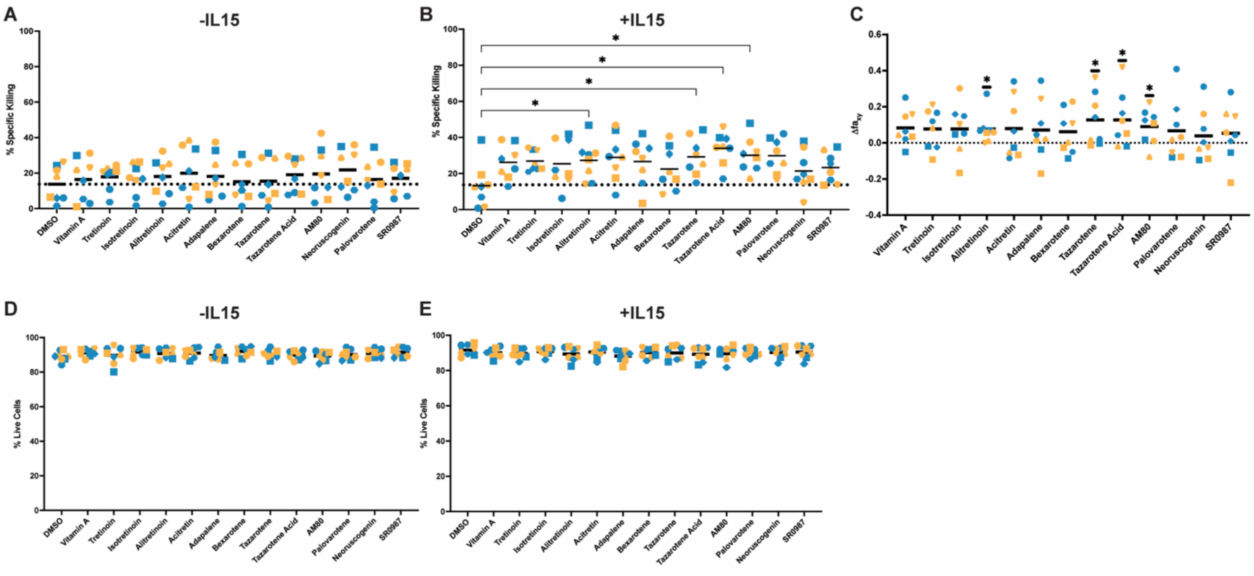

Supplemental Figure 2. **Retinoid enhance killing of HIV-infected cells**. Analysis of percent specific killing in the absence (**A**) or presence (**B**) of IL15. Data was normally distributed resulting in parametric analysis. A one-way ANOVA with multiple comparisons was used to determine significance to the DMSO. (*p<0.05, **p<0.01, ***p<0.001). (**C**) Bliss independence model was used to measure synergistic relationships between the retinoids and IL15. We used a one sample T-test to determine significance (n=8). Toxicity of each retinoid in uninfected CD4T cells in the absence (**D**) or presence of IL15 (**E**). Each participant is designated by their own symbol in each graph. Gold symbols represent male donors, and blue symbols represents female donors.

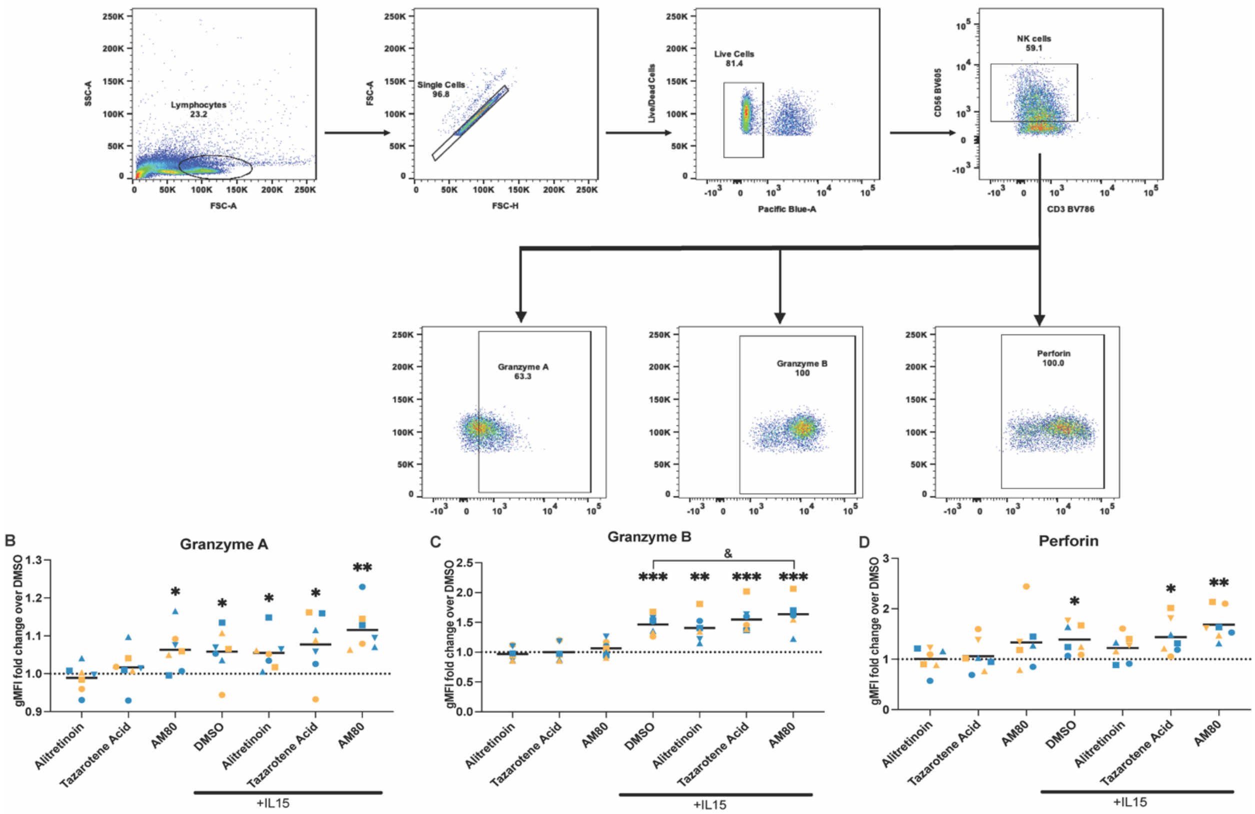

Supplemental Figure 3. IL15 enhances Granzyme A, B and Perforin expression in NK cells. (**A**) Gating strategy. Levels of Granzyme A (**B**), Granzyme B (**C**) and Perforin (**D**) in NK cells cultured with 1μM of each retinoid with or without IL15. Data was normally distributed resulting in parametric analysis. A one-sample T test was used to measure fold change compared to the DMSO control (*$p<0.05$, **$p<0.01$, ***$p<0.001$). The combination of retinoids with IL15 was compared to DMSO with IL15 using the Dunnet's multiple comparison test ($^{\&}p<0.05$). Each participant is designated by their own shape in each graph. Gold symbols represent male donors, and blue symbols represent female donors.

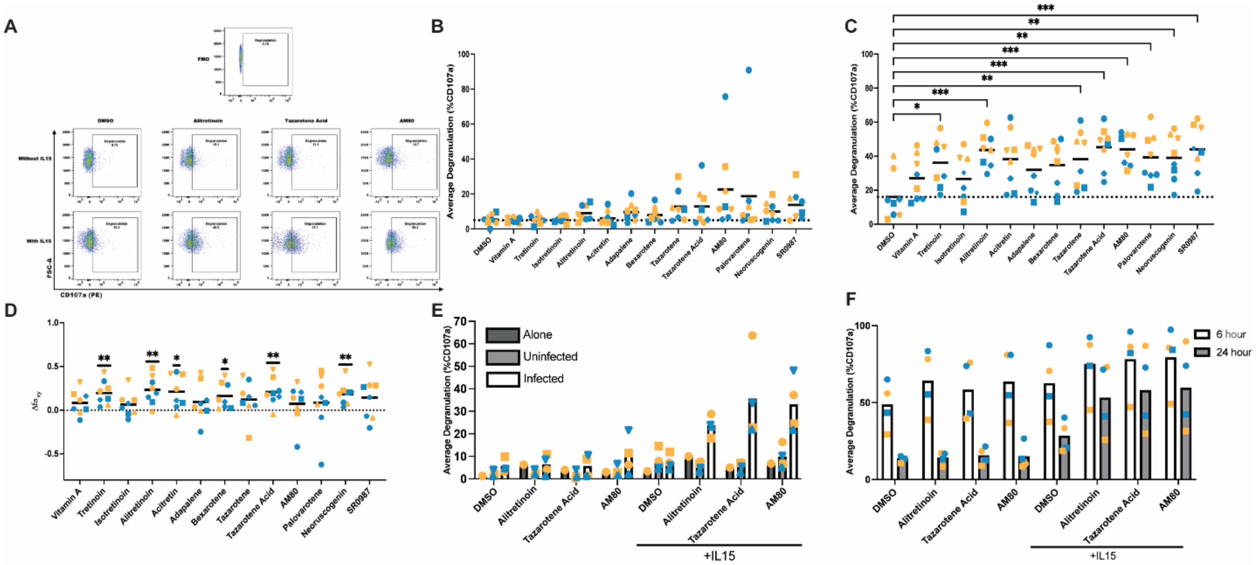

Supplemental Figure 4. **Retinoids enhance NK degranulation.** (**A**) Representative flow gating. Analysis of NK degranulation in the absence (**B**) or presence (**C**) of IL15 with the addition of retinoids. Data was normally distributed resulting in parametric analysis. A one-way ANOVA with multiple comparisons was used to determine significance to the DMSO (*p<0.05, **p<0.01, ***p<0.001, ****p<0.0001). (**D**) Bliss independence model was used to measure synergistic relationships between the retinoids and IL15. We used a one sample T-test to determine significance (n=8). (**E**) We compared degranulation with NK cells cultured alone, with uninfected CD4 T cells and with HIV-infected cells (n=4). (**F**) Average degranulation of NK cells at 6 hours and 24 hours. Each participant is designated by their own shape in each graph. Gold symbols represent male donors, and blue symbols represent female donors.

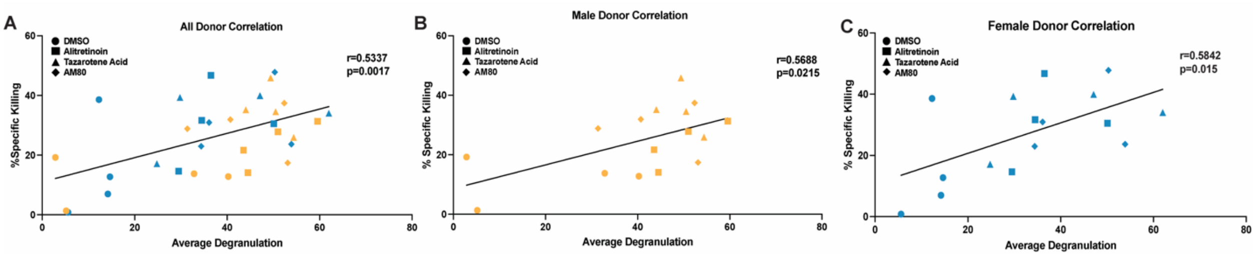

Supplemental Figure 5. Specific killing and NK degranulation are positively correlated. Data was normally distributed resulting in parametric analysis. Pearson correlation between average degranulation and percent specific killing for (**A**) all donors and then separated them out by (**B**) male (n=4) and (**C**) female (n=4) donors.

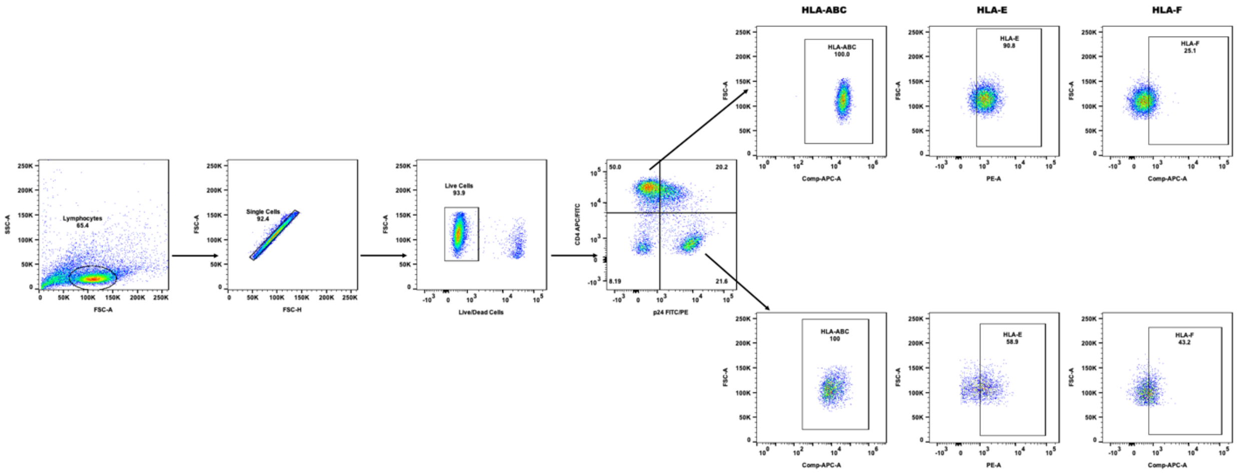

Supplemental Figure 6. Gating strategy for MHC-I expression for Figure 3.

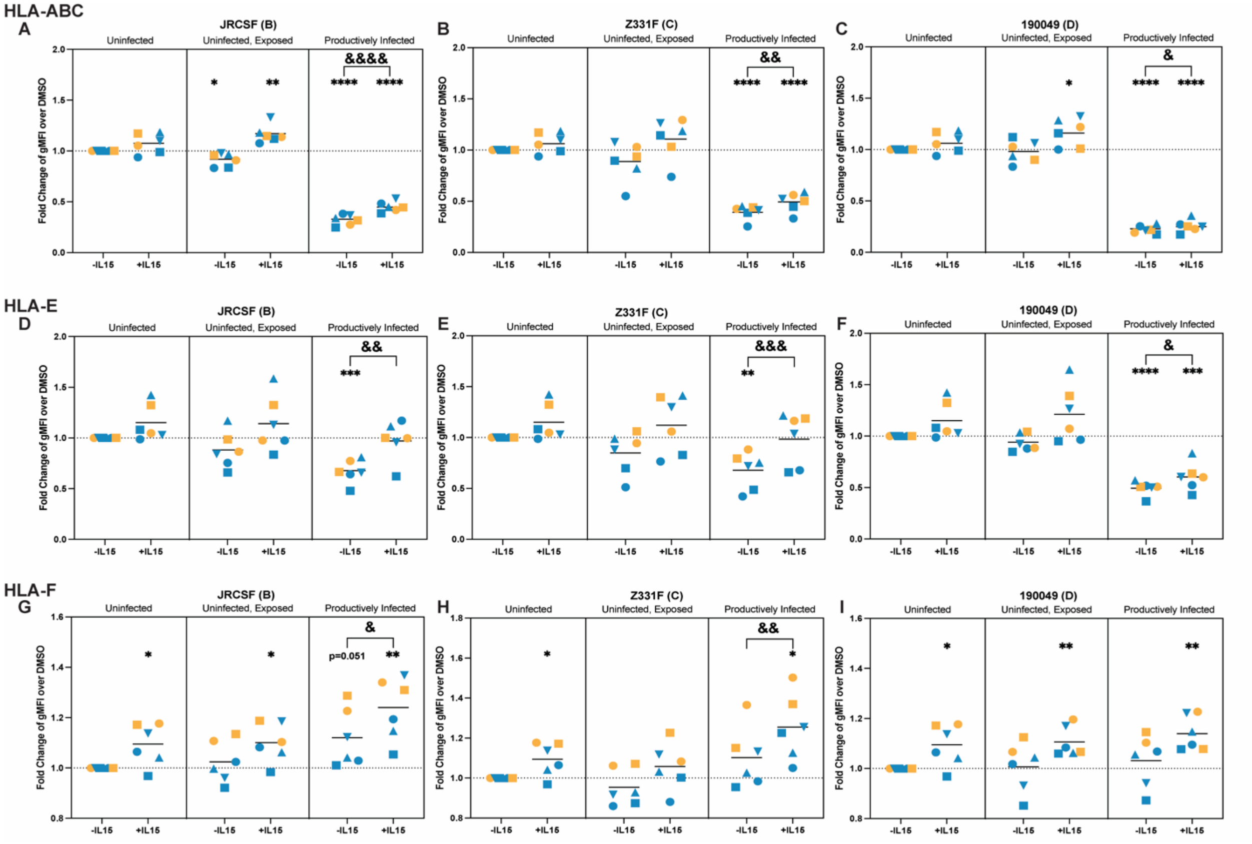

Supplemental Figure 7. Changes in HLA expression are similar across HIV subtypes. Fold change expression of geometric mean intensity fluorescence (gMFI) over DMSO of uninfected and infected cells. Analysis of (**A, B, C**) HLA-ABC (n=6), (**D, E, F**) HLA-E (n=6) and (**G, H, I**) HLA-F (n=6) of CD4 T cells infected with JRCSF, Z331F and 190049. Data was normally distributed resulting in parametric analysis. A one sample T test was used to compare the fold change to the DMSO of uninfected cells, (*p<0.05, ****p<0.0001) and a paired t-test was used to compare the addition of IL15 to the absence of IL15 in HIV infected cells ($^{\&}$p<0.05, $^{\&\&}$p<0.01, $^{\&\&\&}$p<0.001). Each participant is designated by their own symbol in each graph. Gold symbols represent male donors, and blue symbols represent female donors.

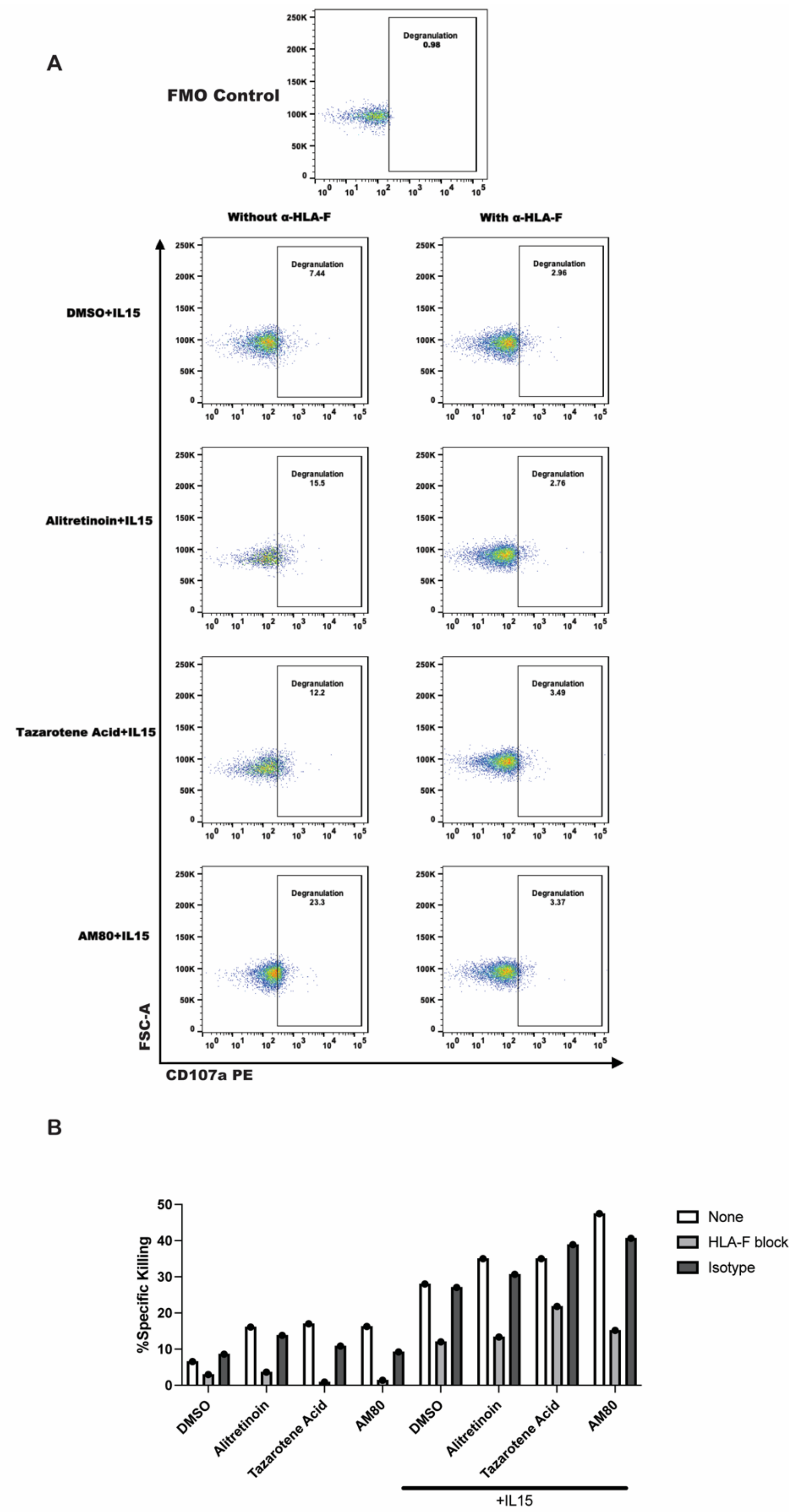

Supplemental Figure 8. **Anti-HLA-F blocks degranulation and killing.** (**A**) Gating strategy for degranulation for Figure 3B. (**B**) HLA-F blocking antibody compared to Mouse IgG1 Isotype control or no antibody.

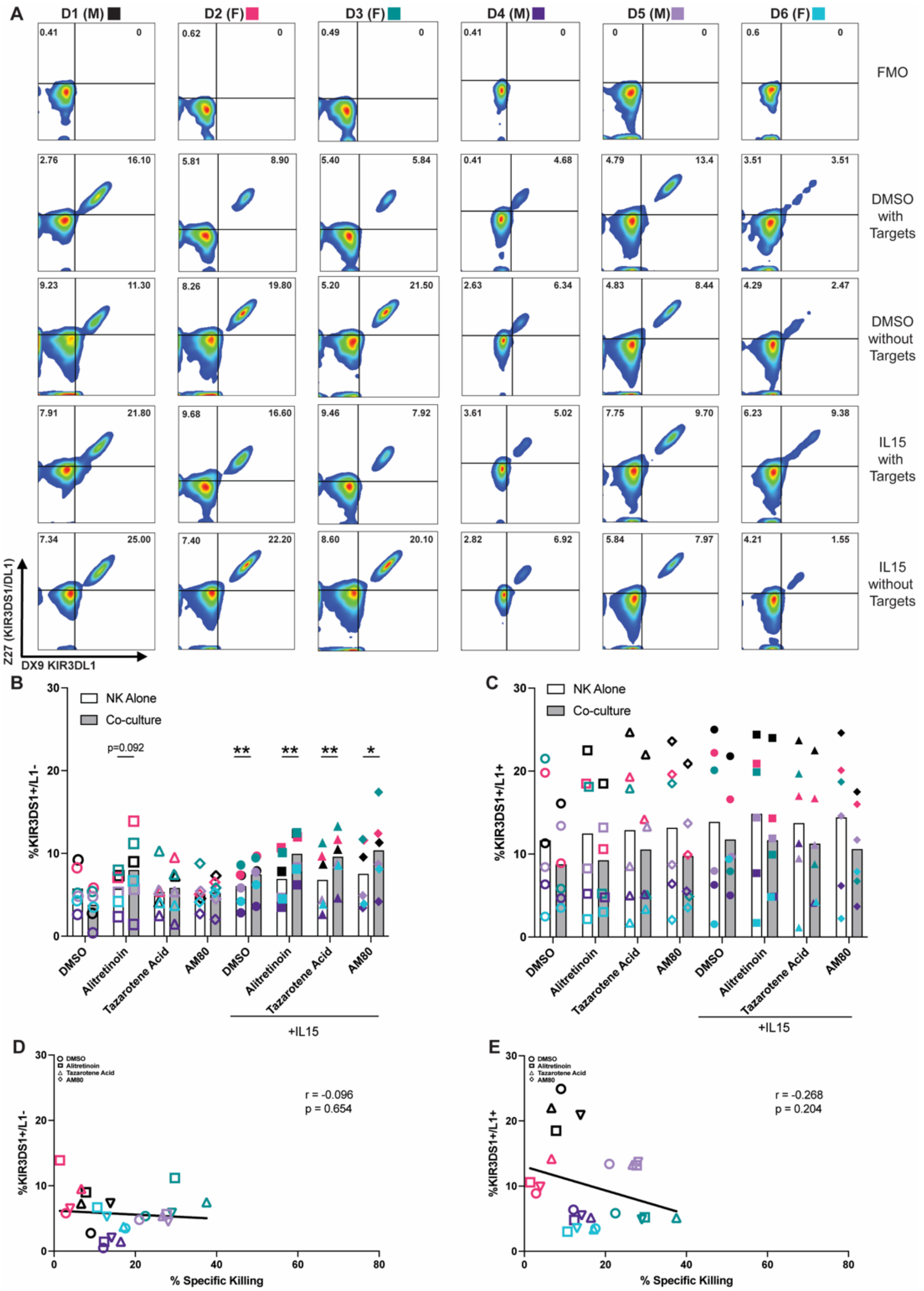

Supplemental Figure 9. Evaluation of KIR3DS1+/KIR3DL1- and KIR3DS1+/KIR3DL1+. (**A**) Flow gating to measure both KIR3DS1+/KIR3DL1- and KIR3DS1+/KIR3DL1+ in each of the donors. Correlation analysis in the absence of IL15 of Percent Specific Killing with (**B**) KIR3DS1+/KIR3DL1- and (**C**) KIR3DS1+/KIR3DL1+. Comparison of NK cells alone to co-cultured with target cells to measure (**D**) KIR3DS1+/KIR3DL1- and (**E**) KIR3DS1+/KIR3DL1+. Data was normally distributed resulting in parametric analysis. A paired T test was used to compare to the absence and presence of targets (*$p<0.05$). Symbols coincide with treatment with open symbols representing the absence of IL15 and closed symbols representing the presence of IL15. Each participant was represented by a different color.

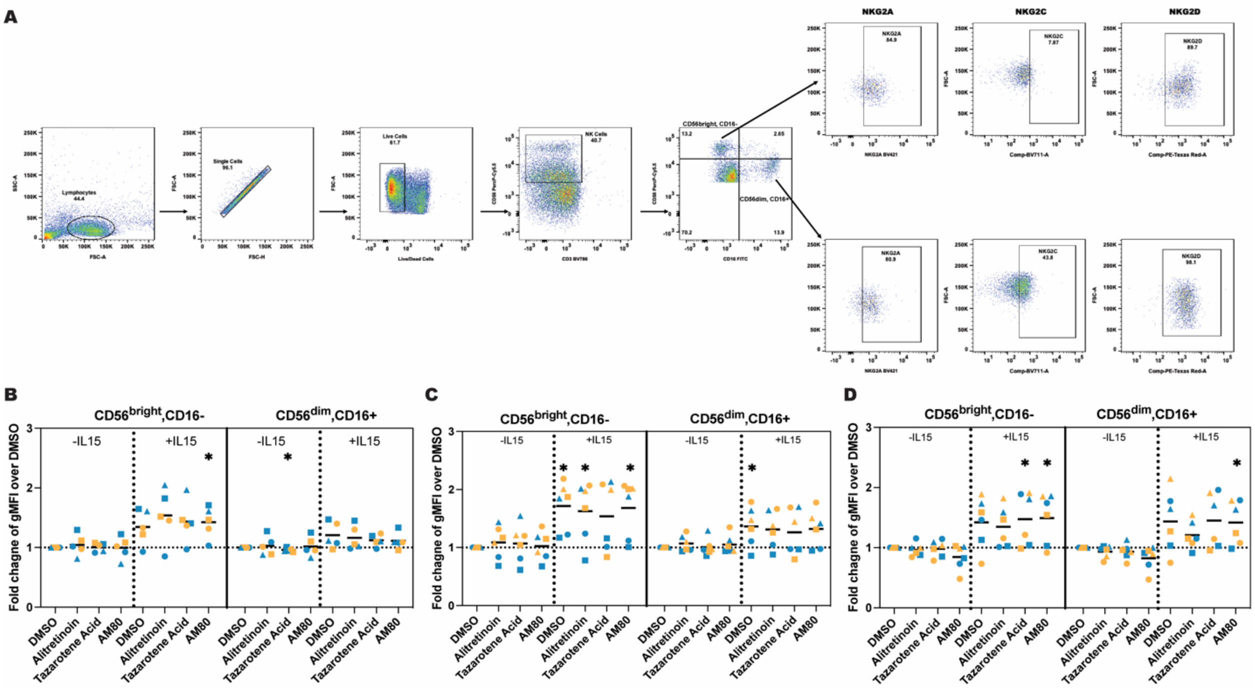

Supplemental Figure 10. Retinoids do not influence NKG2 expression in NK cells. (**A**) Flow gating strategy to identify NKG2 expression in the cytokine producing (CD56^{bright}, CD16-) and the cytotoxic (CD56^{dim}, CD16+) NK cells. (**B**) NKG2A (n=5), (**C**) NKG2C (n=6) and (**D**) NKG2D (n=6). Data was normally distributed resulting in parametric analysis. A two-way ANOVA with multiple comparisons was used to determine significance (*p<0.05). Each participant is designated by their own symbol in each graph. Gold symbols represent male donors, and blue symbols represent female donors.

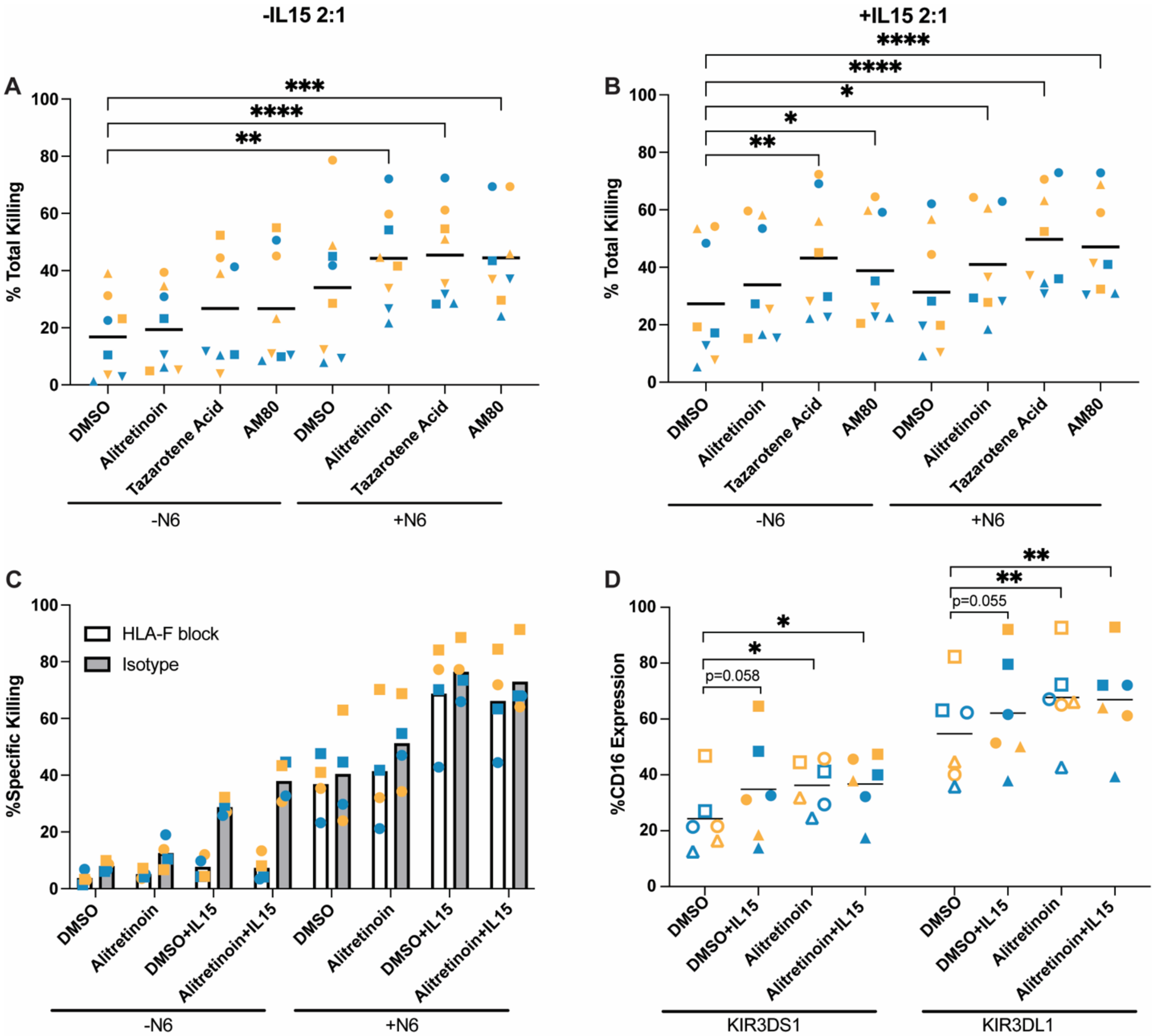

Supplemental Figure 11. Retinoids enhance ADCC in an HLA-F independent manner. Total killing of HIV infected CD4 T cells with the addition of the N6 antibody and retinoids in (**A**) the absence or (**B**) presence of IL15 with an E:T ratio of 2:1 (n=8). Data was not normally distributed resulting in non-parametric analysis. The Friedman test was used to compare the percent total killing in the presence of the N6 antibody to the DMSO control in the absence of the N6 antibody (*p<0.05, **p<0.01, ***p<0.001, ****p<0.0001). (**C**) ADCC in the presence of blocking HLA-F blocking (n=4). (**D**) Analysis of CD16 expression (n=6) in both KIR3DS1+ and KIR3DL1+ NK cells. A two-way ANOVA was used to compare the percent of CD16 expression to the DMSO for each NK cell population (*p<0.05, **p<0.01). Each participant is designated by their own shape in each graph. Blue represents female and gold represents male donors.

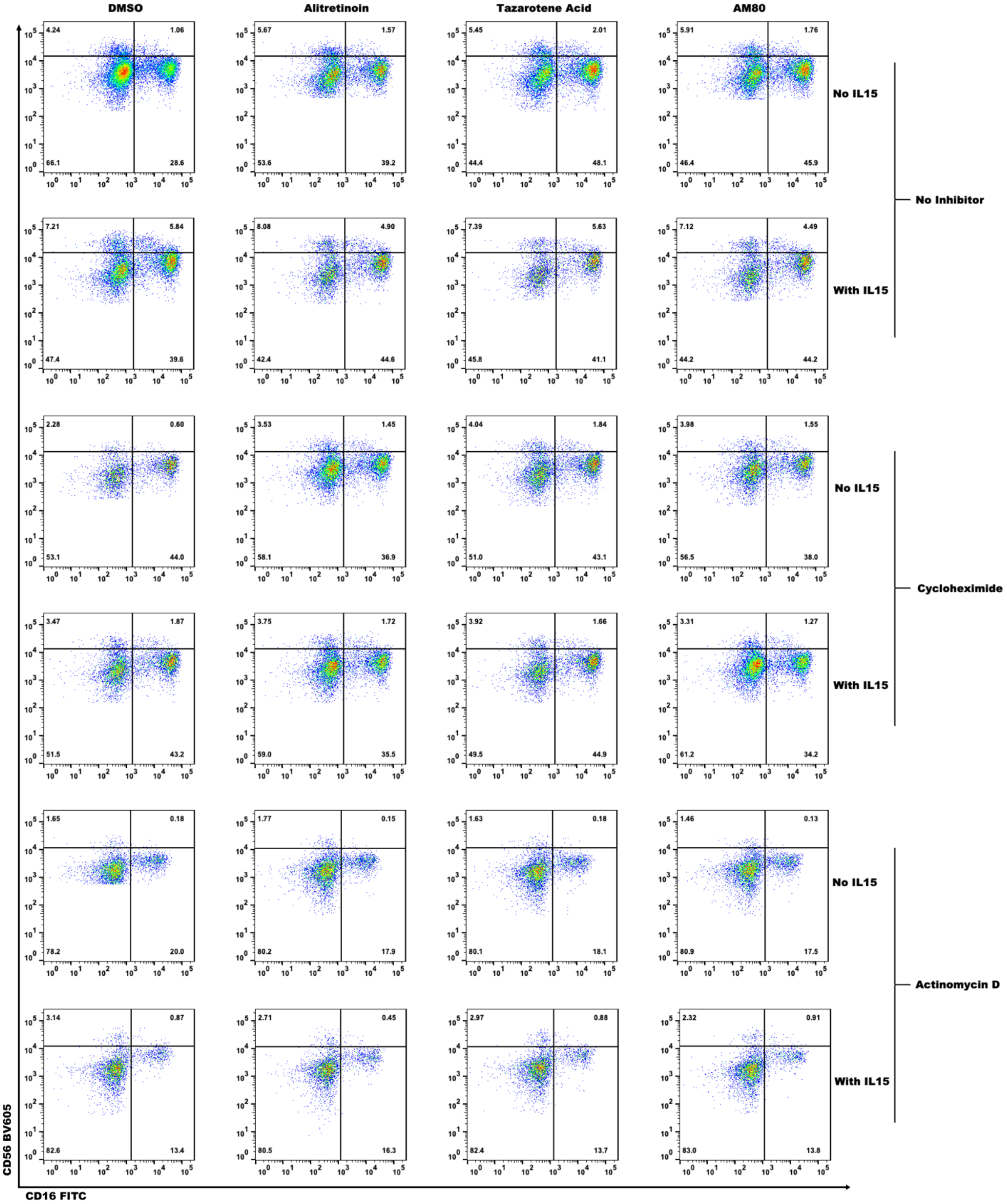

Supplemental Figure 12. Gating strategy for CD16 expression for Figure 4E.
